# What does not kill you makes you stronger? Effects of paternal age at conception on fathers and sons

**DOI:** 10.1093/evolut/qpae097

**Published:** 2024-09-03

**Authors:** Krish Sanghvi, Tommaso Pizzari, Irem Sepil

**Affiliations:** Department of Biology, University of Oxford, Oxford, United Kingdom

**Keywords:** senescence, viability selection, Lansing effect, terminal investment, life-history trade-offs, pleiotropy

## Abstract

Advancing male age is often hypothesized to reduce both male fertility and offspring quality due to reproductive senescence. However, the effects of advancing male age on reproductive output and offspring quality are not always deleterious. For example, older fathers might buffer the effects of reproductive senescence by terminally investing in reproduction. Similarly, males that survive to reproduce at an old age might carry alleles that confer high viability (viability selection), which are then inherited by offspring, or might have high reproductive potential (selective disappearance). Differentiating these mechanisms requires an integrated experimental study of paternal survival and reproductive performance, as well as offspring quality, which is currently lacking. Using a cross-sectional study in *Drosophila melanogaster*, we test the effects of paternal age at conception (PAC) on paternal survival and reproductive success, and on the lifespans of sons. We discover that mating at an old age is linked with decreased future male survival, suggesting that mating-induced mortality is possibly due to old fathers being frail. We find no evidence for terminal investment and show that reproductive senescence in fathers does not onset until their late-adult life. Additionally, we find that as a father’s lifespan increases, his probability of siring offspring increases for older PAC treatments only. Lastly, we show that sons born to older fathers live longer than those born to younger fathers due to viability selection. Collectively, our results suggest that advancing paternal age is not necessarily associated with deleterious effects for offspring and may even lead to older fathers producing longer-lived offspring.

## Introduction

Reproductive senescence is the age-dependent decline in the reproductive output of organisms (Monaghan et al., 2008; Rose & Charlesworth, 1980). This leads old individuals to have lower gamete quality and quantity (Dean et al., 2010; Gasparini et al., 2010), thus lower fecundity and fertility (Naciri et al., 2022; Sepil et al., 2020), and produce fewer offspring, than young individuals. For instance, old males across different species often have smaller ejaculate sizes (Cornwallis et al., 2014; Sanghvi et al., 2024), poorer quality sperm (Gasparini et al., 2019; Johnson et al., 2015), and lower abundance of seminal fluid proteins (Fricke et al., 2023), than young males. A growing body of literature however, challenges these patterns by documenting that advancing male age does not necessarily result in male reproductive senescence (Aich et al., 2022; Baudisch & Stott, 2019; Brooks & Kemp, 2001; Cooper et al., 2020, 2021; Finch, 2009; Forslund & Pärt, 1995; Heinze et al., 2018; Johnson & Gemmell, 2012; Jones & Vaupel, 2017; Lee & Chu, 2023; Moullec et al., 2023; Sandfoss et al., 2023; Segami et al., 2021; Vega-Trejo et al., 2019). In some cases, advancing age may even be associated with increased reproductive output (e.g., Avent et al., 2008; Girndt et al., 2019; Lifjeld et al., 2022; Prathibha et al., 2011; Sanghvi et al., 2024; Santhosh & Krishna, 2013; Verspoor et al., 2015).

Several mechanisms explain why old males might have a reproductive output similar to-, or higher than, young males. At a population level, between-individual heterogeneity in quality might lead to the selective disappearance of poor-quality individuals. Here, longer-living males could represent a nonrandom cohort with higher reproductive quality (Bouwhuis et al., 2009; Hämäläinen et al., 2014; Sanghvi et al., 2022; Sultanova et al., 2020), thus masking reproductive senescence. Alternatively, according to the terminal investment hypothesis, old males, males who perceive themselves as close to dying, or old males close to dying, might allocate proportionally more resources, such as ejaculates, to a single current reproductive event than young males (Duffield et al., 2017; Farchmin et al., 2020; Moullec et al., 2023). This is because as future survival prospects of organisms decline, individuals are hypothesized to invest more in current than future reproductive opportunities (Creighton et al., 2009; Duffield et al., 2018; Froy et al., 2013; Part et al., 1992; Velando et al., 2006).

In addition to impacting a male’s own reproductive output, reproductive senescence in males can also influence the phenotypes of the offspring via paternal age effects (Priest et al., 2002; Schroeder et al., 2015). For instance, offspring sired by old fathers are reported to have slower development and more developmental disorders (Janecka et al., 2017; Preston et al., 2015), poorer early life performance (Fay et al., 2016) and reproductive output (Arslan et al., 2017; Nystrand & Dowling, 2014; Vuarin et al., 2021), than offspring of young fathers. Notably, offspring born to old fathers often have shorter lifespans than those born to young fathers (Crow, 2003; Monaghan et al., 2020; Sharma et al., 2015), a phenomenon known as the “Lansing effect” (Ivimey-Cook et al., 2023; Lansing, 1947). While these paternal age effects might be caused by age-dependent changes in paternal care (Benowitz et al., 2013; Cope et al., 2022), in species without care, these effects likely occur via age-dependent deterioration in ejaculates (Monaghan & Metcalfe, 2019) or differential resource allocation by females (Harris & Uller, 2009).

In contrast to the negative effects of old fathers, few studies report no effects of paternal age on offspring quality (Heinze et al., 2018; Sparks et al., 2022). Others yet show that old fathers can produce larger (Aguilar et al., 2023; Mirhosseini et al., 2014), longer lived (Angell et al., 2022; Johnson et al., 2018; Krishna et al., 2012; Lee et al., 2019; Priest et al., 2002), and more fecund (Krishna et al., 2012; Sparks et al., 2022) offspring than young fathers (also see Kroeger et al., 2020 for beneficial maternal age effects). Thus, some mechanisms have been proposed to explain why old fathers produce higher-quality offspring compared to young fathers. Males who mate at an old age are predicted to have, on average, longer lifespans than males who mate at younger ages (Mueller, 2004). If differences in survival between individuals are due to intrinsic reasons, older fathers would carry alleles that confer higher viability (Bowen et al., 2006; Chen & Maklakov, 2012; Kokko & Lindström, 1996; Reznick et al., 2004). Consequently, offspring born to older fathers would inherit these alleles, leading to old fathers producing longer-lived offspring, as predicted by the viability selection hypothesis (Beck et al., 2002; Brooks & Kemp, 2001; Hansen & Price, 1995; Johnson & Gemmell, 2012; Kokko, 1998). However, this positive relationship between paternal age and paternal or offspring lifespan might be buffered when old males are frail and die soon after mating or under high extrinsic mortality (Kokko & Lindström, 1996). In contrast with selective disappearance, where longer-lived individuals have higher reproductive output, the viability selection hypothesis specifically pertains to the selection for higher lifespans with increasing age.

The effects of paternal age at conception (PAC) and paternal lifespan on paternal reproductive output and offspring lifespan are unlikely to be independent. Nonmutually exclusive processes (effects on male reproductive output: *reproductive senescence, selective disappearance, terminal investment*; effects on offspring quality: *Lansing effect, viability selection*) might co-occur and interact with each other to shape the life-history of fathers and their offspring. For example, higher paternal allocation toward producing offspring of high quality could come at the cost of lower allocation toward producing many offspring or fathers living longer due to life-history trade-offs (e.g., Johnson et al., 2018; Lemaître et al., 2015; Travers et al., 2021). Different processes could further interact to shape patterns of aging in animals. For instance, selective disappearance might lead to beneficial effects of advancing age on reproductive output until mid-life. However, from mid- to late-life, these age-dependent improvements could be outweighed or balanced by deleterious effects of senescence, leading to curvilinear shapes of aging (e.g., Cooper et al., 2020; Jones et al., 2014; McCleery et al., 2008; Reid et al., 2010; Sanghvi et al., 2024; Torres et al., 2011), or no overall effects of age (e.g., Cooper et al., 2021). Only a few studies (on wild populations) have attempted to disentangle the confounding effects of PAC and paternal lifespan (e.g., Reid et al., 2010; Torres et al., 2011). While valuable for their ecological relevance, these studies on wild populations have limited control over confounding factors such as maternal aging and male mating history (a crucial modulator of male survival and reproductive output: Aich et al., 2022; Jones & Elgar, 2004; Partridge & Andrews, 1985; Paukku & Kotiaho, 2005).

Using cross-sectional sampling of fathers at different ages in the fruit fly, *Drosophila melanogaster*, we test the effects of PAC on paternal survival (aim 1), paternal reproductive output (aim 2), and the lifespans of sons (aim 3). A cross-sectional design allows us to test whether between-individual variation in lifespan and reproduction; therefore, population-level processes, such as viability selection and selective disappearance, explain the influence of PAC on offspring phenotypes. We first investigate the influence of PAC on paternal lifespan and future survival (aim 1). The relationship between PAC and paternal lifespan is often assumed to be positive. However, this relationship might be nonlinear due to processes not usually accounted for (Supplementary Appendix 1). For instance, males who mate when old might be frail, thus incur more severe costs of mating and die soon after mating than males who mate at a young age. Such age-dependent frailty could lead to a lower ceiling for maximum lifespan in males who mate when old than in males who mate when young, therefore buffering the expected positive relationship between PAC and paternal lifespan. Next, we investigate whether a male’s age at conception affects his reproductive output (aim 2, Supplementary Appendix 2). Consistent with reproductive senescence, old males might have a lower reproductive output than young males. However, between-individual heterogeneity arising when males are sampled cross-sectionally might mask reproductive senescence at a population-level. For example, when male mortality is nonrandom and lifespan and reproductive output covary positively, males with low reproductive output might die earlier than males with high reproductive output. Such selective disappearance would lead to an increase in reproductive output with advancing age. Furthermore, terminal investment could lead old males, males who are close to dying, or old males close to dying, to invest more in a current reproductive event than young males or males far away from dying. Finally, we investigate how PAC affects the lifespans of offspring, focusing on sons (aim 3, Supplementary Appendix 2). Here, in line with Lansing effects, old fathers might produce shorter-lived sons than young fathers. However, if old fathers have alleles that confer longer lifespans, then a cross-sectional sampling of paternal ages could lead to sons born to old fathers living for longer, than those born to young fathers due to viability selection.

## Methods

### Stock population

To investigate how PAC affects paternal survival and reproductive output, as well as the lifespan of sons, we first set up a population of experimental males. To do this, we collected 300 virgin males (henceforth “unmated experimental males”) within 6 hr of eclosion on ice and placed them in individual vials. These 300 males were collected from a stock population cage of lab-adapted, outbred, wild-type Dahomey *D. melanogaster* flies maintained in our lab since the 1970s. Unmated experimental flies were reared using a standard larval density method at 25 °C and 45% relative humidity (RH) (Clancy & Kennington, 2001). All flies in our experiment were maintained on a 12:12 hr light cycle and fed with Lewis medium supplemented with ad libitum live yeast (Lewis, 1960). Under these conditions, male flies in our lab have median and maximum lifespans of ~45 days and ~90 days, respectively.

### Experimental design

We first generated different PAC treatments of experimental fathers by sampling fathers using a cross-sectional design. A cross-sectional sampling approach was employed for two reasons. First, we were interested in testing whether between-individual heterogeneity in paternal lifespan might buffer the deleterious effects of advancing paternal age. Here, only a cross-sectional design could create heterogeneity in paternal lifespan for different PAC treatments. Our second reason for cross-sectional sampling was to prevent paternal age from confounding with paternal mating history, which inevitably occurs when fathers are sampled longitudinally (Aich et al., 2022).

Every two weeks (starting at 4 days of age), we chose between 25 and 37 surviving males from the 300 unmated experimental males (sample sizes in Supplementary Table S1). In total, we generated a total of six PAC treatments, with males of the following ages (in days): 4, 18, 32, 46, 60, and 74 (see Supplementary Table S1 for sample sizes). We generated random numbers in MS Excel (“randbetween” function) to randomize our choice of males. The chosen experimental males were placed in a vial with a young (4-day-old) virgin female of Dahomey background. Chosen experimental males were mated only once with the female, and males that did not mate within 4 hr (five in total across all PAC treatments) were censored. Each mating was observed, and mating latency and copulation duration were recorded. After mating, the males were transferred into a new vial and kept individually until they died, and monitored daily to record their lifespans within 1 day of error. All fathers in our experiment mated only once across their lifespan. The mated females were left in the mating vial for another 24 hr following exposure to the male to lay eggs, after which the females were discarded. These vials were left at 25 °C and 45% RH for 9–10 days until offspring emerged (pupae in all vials emerged).

Within 6 hr of emergence, three sons were selected haphazardly from each parental pair and moved into a separate vial. All three sons from a parental pair were kept together until death. The remaining offspring from each parental pair were frozen at −20 °C on the fourth day after eclosion began, and their numbers were counted. We additionally recorded the survival of the unmated experimental males each day to compare the survival of mated versus unmated males. All experimental flies, including unmated males and sons, were put in new food vials once every 4 days using an aspirator.

### Data analysis

We used R v3.5.2 for all analyses (see HTML supplement for code and full model outputs), and packages *lme4* (Bates et al., 2015), *glmmTMB* (Brooks et al., 2017), *lavaan* (Rosseel, 2012), and *nlme* (Pinheiro et al., 2017) for building models. The values of *p* for all linear models were calculated using Satterthwaite’s method. All linear models were checked for model assumptions of normality and homoscedasticity of residuals using the *stats* package (R Core Team, 2012). All generalized linear models were checked for overdispersion using *DHARMa* (Hartig & Hartig, 2017). All Cox proportional hazards models were checked for the assumption of constant hazard ratio over time for each fixed effect, using the cox.zph function in the survival package, which performs tests on Schoenfeld residuals. We interpreted the main effects only when two-way interactions were nonsignificant (following Engqvist, 2005) by fitting a model with the interaction removed. PAC was modeled as a continuous, not categorical variable, in all our models.

### Aim 1: Effects of PAC on paternal survival

First, we investigated the effects of increasing PAC on paternal lifespan (see Supplementary Appendices 1 and 2 for hypotheses and predictions). For this, we created a linear model with PAC as a fixed effect and paternal lifespan as the dependent variable. Our data violated linear model assumptions of homogenous variance due to lower variance in paternal lifespans for older PAC treatments. Thus, we specified a heterogeneous variance structure using the function *gls* (Pinheiro et al., 2017). A model with heterogenous variance structure was a better fit to the data than one without (ΔAIC = 7.817; ΔDF = 5).

Second, we tested whether mortality risk differed between mated (i.e., fathers) and unmated experimental males. For this, we compared survival probability between unmated experimental males and fathers who mated when young (4 days old). Young rather than older fathers were chosen for this comparison because we wished to compare actuarial senescence patterns across the entire lifetime of mated versus unmated males. Using old fathers would not allow this comparison because older fathers, by definition, survived to older ages. We modeled data using Cox proportional hazards in the *coxme* (Therneau & Therneau, 2015) and *survival* (Therneau & Lumley, 2013) packages, with male lifespan as the dependent variable and treatment (4 days old mated versus unmated) as a fixed effect.

Third, we compared whether mortality risk specifically due to mating, differed between young and old males. For this, we used two approaches. In our first approach, we used a generalized linear model with binomial error distribution. Here, we compared the proportion of males that were alive (1) or dead (0) as our dependent variable in each PAC treatment (fixed effect), 3 days after mating. Here, we additionally included the number of offspring each male produced as a fixed effect to test for potential trade-offs between paternal mortality risk and offspring production (Lemaître et al., 2015). However, differences in survival soon after mating between different PAC treatments could, in principle, be explained by actuarial senescence alone. We thus used a second approach to specifically test whether mortality risk differed between old and young males due to mating per se by removing the effects of actuarial senescence. To do this, we used a Cox proportional hazards model, where we compared survival probabilities between males who mated when 60 days (i.e., old PAC) old and males mated when 4 days old (i.e., young PAC), while only using data of males from both PAC treatments that survived beyond 60 days. Similarly, we also compared survival probabilities between males who mated when 74 days old versus 4 days old, but only using data on males from both PAC treatments that survived beyond 74 days. Out of the 25 fathers initially assigned to the 4-day-old treatment, 14 (56%) and 6 (24%) survived beyond 60 and 74 days, respectively.

### Aim 2: Effects of PAC on paternal reproductive output

We tested the effect of PAC on paternal probability of siring an offspring (*p*_*s*_), and on the number of offspring sired (*N*_*s*_) when only considering fathers who sired offspring. To do this, we used a hurdle generalized linear model with truncated negative binomial error distribution (Brooks et al., 2017) and number of offspring as our dependent variable. A hurdle model allowed us to first compare the *p*_*s*_ of fathers (i.e., zero inflation model). Then, using only data on fathers that sired offspring (conditional model), compare the (*N*_*s*_) of fathers. PAC, paternal lifespan, their interaction, a quadratic effect of PAC, paternal copulation duration, and mating latency were included as fixed effects. PAC was included to test for senescence, while paternal lifespan was included to test for selective disappearance (van de Pol & Verhulst, 2006). Copulation duration was included to account for males potentially producing more offspring due to transferring more sperm by copulating for longer (Dore et al., 2021). Mating latency was included to account for differences in mate choice because shorter latencies might be due to females preferring the presented male, which could influence how many offspring they produce. In the same model, we also included a quadratic term for PAC, to test for curvilinear patterns of aging. This model would allow us to test for the effects of reproductive senescence, as well as selective disappearance, by testing whether within each PAC treatment, fathers with longer lifespans sired more offspring than fathers with shorter lifespans. We conducted a sensitivity analysis to ensure that the patterns observed for reproductive aging were not driven by the oldest age group (i.e., 74-day-old males) by reanalyzing the data but excluding the 74-day-old males, as this age group had few data points (Supplementary Table S1).

We then investigated whether observed patterns in paternal reproduction could be due to terminal investment. Terminal investment could occur solely as a response to advancing male age, proximity to death, or due to an interaction between male age and proximity to death (reviewed in Duffield et al., 2017; Foo et al., 2023). We thus first tested whether old fathers who were close to dying produced more offspring than old fathers not close to dying or young fathers. For this, we calculated the number of days elapsed between a male’s death and the day he mated, henceforth called “days to death.” Then, using a generalized linear model with zero-inflated negative binomial error distribution, we tested how the interaction between days to death and PAC (fixed effects) affected the number of offspring produced (using all data, including fathers who produced zero offspring) by fathers (dependent variable). Here, we additionally included paternal copulation duration as a fixed effect. We then tested whether fathers who were close to dying or fathers who were old produced more offspring than fathers who were not close to dying or young fathers, respectively, by removing the interaction term in the model specified above.

### Aim 3: Effects of PAC on the lifespans of sons

We investigated the effects of PAC on the lifespans of sons and whether the observed effects were due to fathers in older PAC treatments living longer, as predicted by the viability selection hypothesis. For this, we used three approaches. In our first approach, we tested how PAC affected the lifespans of sons (dependent variable) using a linear model. Here, we included PAC as the fixed effect and paternal ID as a random effect. We also included the number of offspring produced as fixed effect to test for trade-offs between paternal investments in offspring lifespan versus number (Johnson et al., 2018). Our models had heterogenous variance structure specified, as this structure yielded a better fit to the data than a model without heterogenous variance (ΔAIC = 25.66; ΔDF = 1).

In our second approach, we tested whether PAC had a significant effect on the lifespans of sons after isolating the variance explained by paternal lifespan. This was done to test whether the effect of PAC on the lifespans of sons, observed in our first approach, was mediated by paternal lifespan (i.e., indirectly) as predicted by the viability selection hypothesis. We first built a model with paternal lifespan as a fixed effect, paternal ID as a random effect, and the lifespan of each son as our dependent variable. Then, we used residuals from this model as the dependent variable and included PAC and the number of offspring produced by fathers as fixed effects, and paternal ID as a random effect. Such a method of residualizing allowed us to test the effects of a PAC while isolating the effects of paternal lifespan, which was a collinear predictor (Dormann et al., 2013). This model had a heterogeneous variance structure specified as described above.

In our third approach, we conducted a path analysis using a structural equation model in the package *lavaan* (Rosseel, 2012). This approach was used to obtain a better understanding of the path of causality for how PAC affects lifespans of sons. We modeled the direct effect of PAC on the son lifespan and its indirect effect via the influence of paternal lifespan. In this model, we also included covariances between the number of offspring produced by fathers with paternal lifespan and with lifespans of sons to account for trade-offs between offspring quality and number and between investment in reproduction versus survival by fathers. For all three approaches used for analyzing the effects of PAC on son’s lifespans, we additionally conducted sensitivity analyses. In these, we excluded data on sons from the 74-day PAC treatment owing to the small sample size of this treatment.

## Results

### Aim 1: Effects of PAC on paternal survival

**Figure 1:**
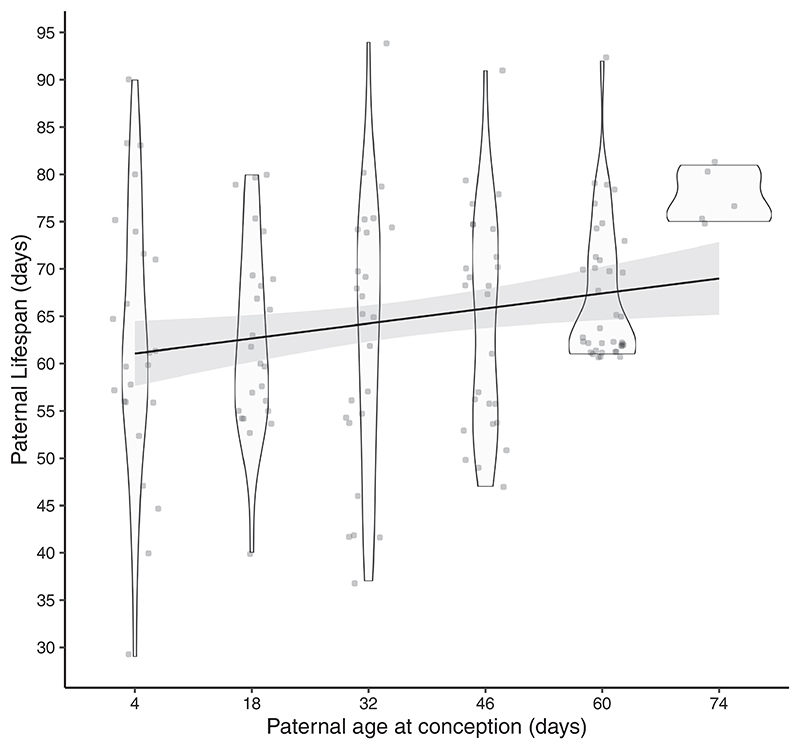
Males who conceive at older ages have longer lifespans on average. Violin plots show the smoothed distribution of data, while dark lines and shaded areas show regression lines and 95% CI. Lower Y-axis limit set to 25 because no deaths occurred before 25 days of age. The sample size of fathers across the six PAC treatments (from 4 to 74 days) is 25, 25, 26, 26, 37, 5, respectively.

Increased PAC was associated with an increase in the average lifespan of fathers (*t* = 3.072; *p* = 0.003, *R*^2^ = 4.3%, Figure 1). Paternal lifespan increased by 0.125 days with an increase of one day in PAC.

**Figure 2:**
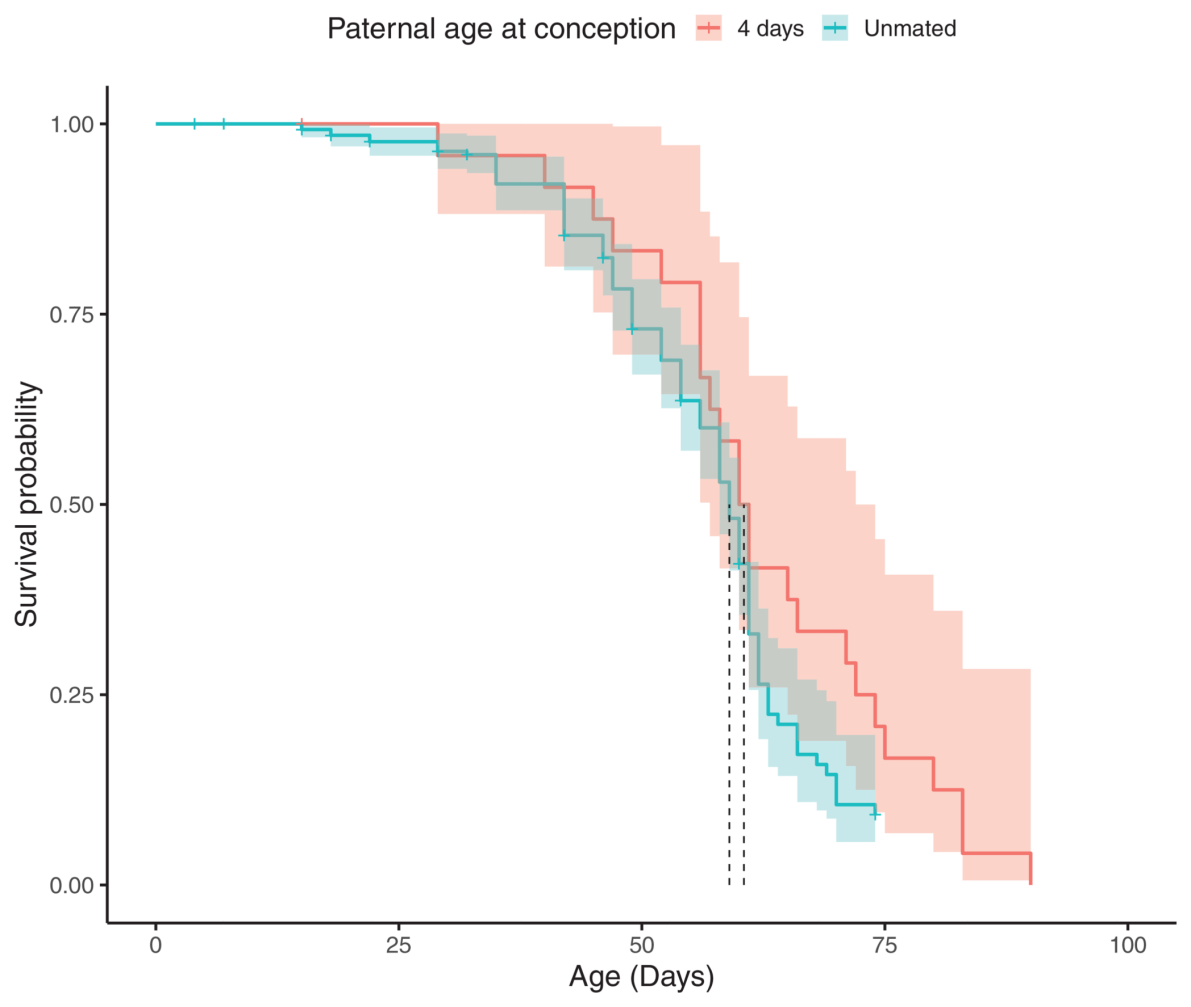
Males who mated at 4 days of age (*N* = 25) did not have a significantly different survival probability compared to the unmated experimental males (*N* = 300). “+” indicates censored individuals. The shaded area shows 95% CI. Dotted lines show age at median survival probability.

**Figure 3:**
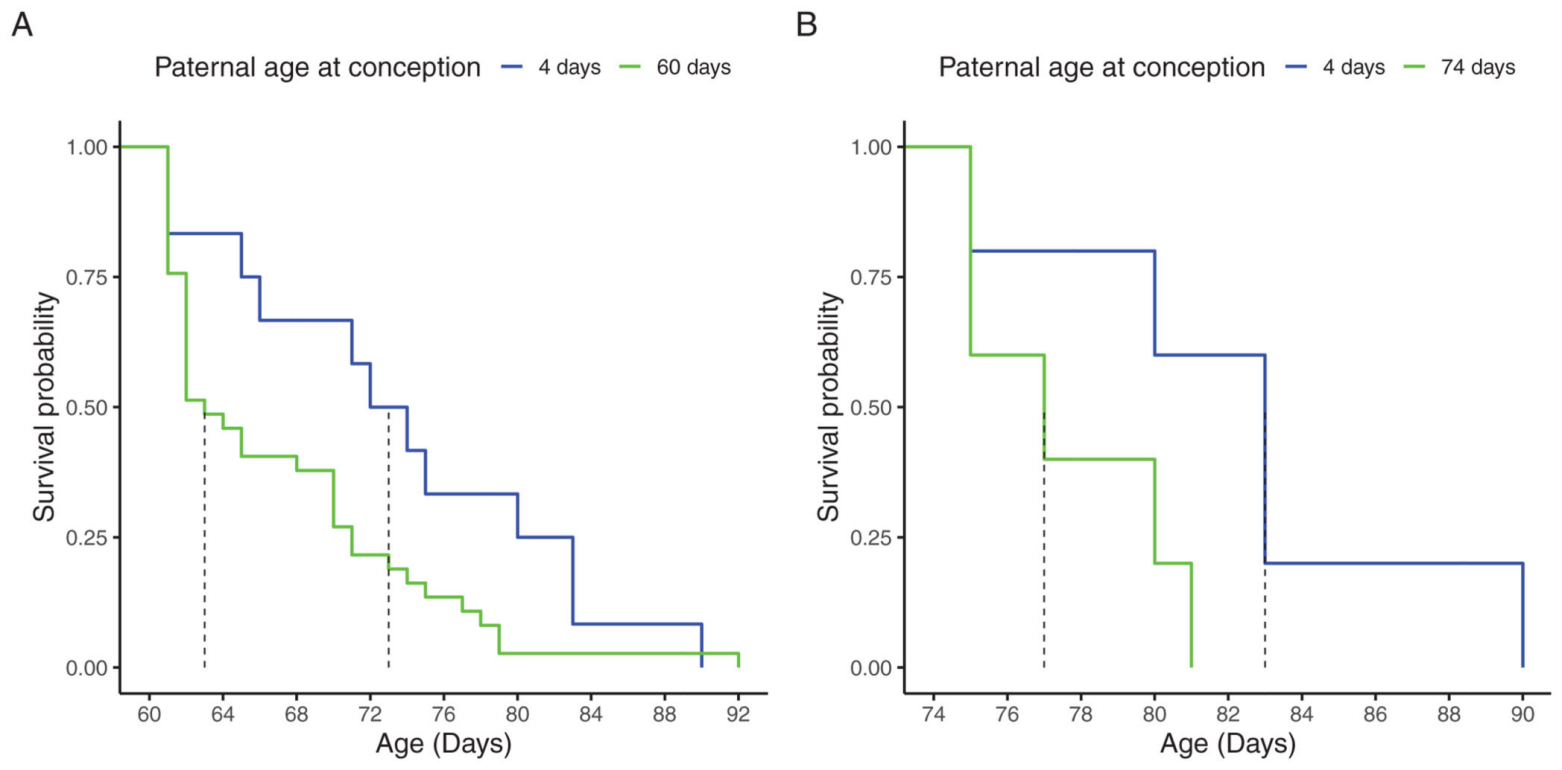
(A) Males who mated at 60 days of age (*N* = 37) had a lower survival probability than males who mated at 4 days of age but survived beyond 60 days of age (*N* = 14). (B) Males who mated at 74 days of age (*N* = 5) had a lower survival probability than males who mated at 4 days of age but survived beyond 74 days of age (*N* = 6). The dotted lines show age at median survival probability.

There was no significant difference in survival probabilities between the unmated experimental males and those who mated at 4 days old (*z* = 1.574, *p* = 0.115, Figures 2 and 3A). When comparing only males who mated, males in older PAC treatments had a lower probability of surviving beyond 3 days after mating than males in younger PAC treatments (*z* = −3.544, *p* < 0.001, Supplementary Table S2; Supplementary Figure S1). For instance, within 3 days after mating, males who mated when “old” (i.e., at 60 or 74 days old) experienced > 50% mortality, compared to no deaths in “young” PAC treatments (i.e., 4, 18, and 32 days old). While this result could be due to age-specific effects of mating stress, it could also be a consequence of actuarial senescence, whereby older PAC have an overall lower age-dependent survival probability than young PAC treatments (*z* = −2.067, *p* = 0.038, Supplementary Figure S2). We thus further tested whether the difference in mortality rates between old and young PAC treatments was specifically due to mating. Males who mated at 60 days of age had a significantly lower probability of surviving past 60 days of age than males who mated at 4 days of age when only data on males that survived past 60 days of age in both PAC treatments were used (*p* = 0.045, Figure 3A). Similarly, males who mated at 74 days of age were less likely to survive past 74 days of age than males who mated at 4 days of age (*p* = 0.066, Figure 3B). Collectively, these results indicate that while advancing PAC is associated with fathers having longer lifespans, the strength of this effect is buffered by old fathers experiencing higher mortality associated with mating stress compared to fathers who mated when young.

### Aim 2: Effects of PAC on paternal reproductive output

**Figure 4:**
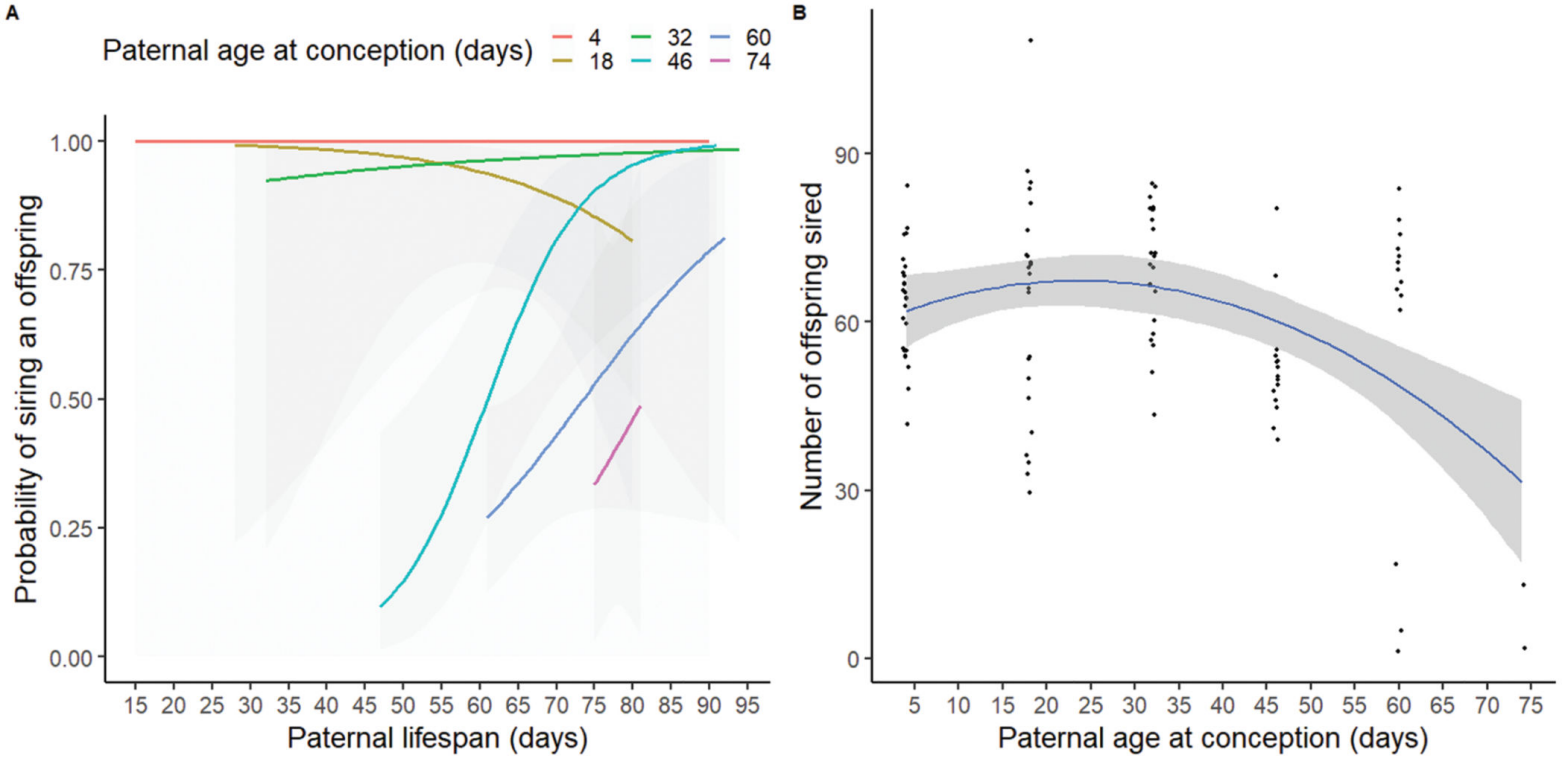
(A) PAC interacted with paternal lifespan to affect the probability of siring an offspring (*p*_*s*_). (B) PAC affected the number of offspring sired (*N*_*s*_: after excluding fathers who did not produce offspring) in a quadratic way. Shaded areas represent 95% CI. Sample size of fathers across the six PAC treatments (from 4 to 74 days) is 25, 25, 26, 26, 37, and 5, respectively.

We found a significant interaction between PAC and paternal lifespan on the probability of siring offspring (i.e., *p*_*s*_: *z* = −2.336, *p* = 0.020, Supplementary Table S3). Specifically, *p*_*s*_ increased with paternal lifespan, but only for fathers who conceived at older ages (Figure 4A). When the interaction term between PAC and paternal lifespan was removed, there was an overall positive effect of increasing paternal lifespan, leading to a higher *p*_*s*_ (*z* = −2.526, *p* = 0.012). However, a model with the interaction term provided a marginally better fit to the data than a model without this term (ΔAIC = 1.4; ΔDF = 2; *p* = 0.065). When investigating variation in *N*_s_ (i.e., number of offspring sired by fathers who produced at least one offspring), we found no significant effects of the interaction between PAC and paternal lifespan (*z* = 0.562, *p* = 0.574, Supplementary Table S3), and no significant effect of paternal lifespan (*z* = 0.963; *p* = 0.336) on paternal *N*_*s*_. However, we found a significant quadratic effect of PAC on *N*_*s*_ (*z* = −3.902, *p* < 0.001, Figure 4B).

Our sensitivity analysis (i.e., a model without data from 74-day-old PAC) showed a significant quadratic effect of PAC on *N*_*s*_ (*z* = −2.084, *p* = 0.037). However, the shape of PAC on *N*_*s*_ from the sensitivity analysis was shallower (Supplementary Figures S3 and S4) than the original model that included data from 74-day-old males. Furthermore, a model with a quadratic and linear term for PAC was no better fit to the data than a model without the quadratic term (ΔAIC = 0.6; ΔDF = 2; *p* = 0.1). This suggests that a decline in *N*_*s*_ late in life and an overall quadratic effect of reproductive aging, are most likely driven by low *N*_*s*_ in the 74-day PAC treatment.

We found a significant interaction between days to death and PAC to affect the number of offspring sired by fathers. A model with an interaction term between PAC and days to death provided a better fit to the data than a model with the interaction removed (ΔAIC = 2.8, ΔDF = 1, *p* = 0.027). Specifically, old males who died soon after mating sired fewer offspring than old males who died later after mating or than young males (*z* = 2.201, *p* = 0.027; Supplementary Table S4). When averaged across other variables, males who were closer to dying did not produce more offspring than males who were not close to dying (*z* = 0.167, *p* = 0.867). Collectively, these results on reproductive output indicate that reproductive senescence in fathers becomes apparent only in late-adult life, that life-history traits of survival and reproductive output show pleiotropic interactions that depend on the age of the father, and that there is no evidence for terminal investment or selective disappearance.

### Aim 3: Effects of PAC on lifespans of sons

**Figure 5:**
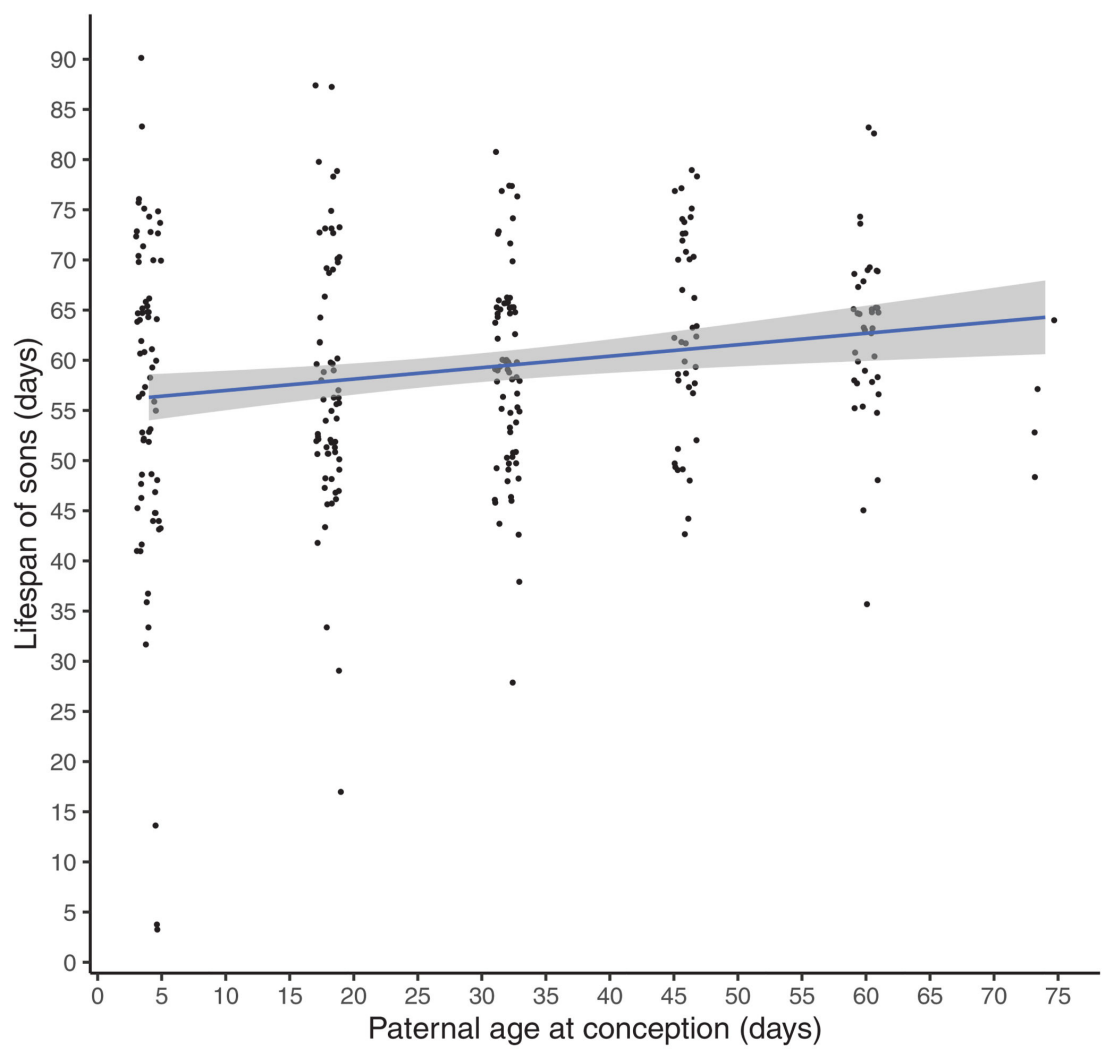
An increase in PAC led to an increase in the lifespans of sons. Each dot represents the lifespan of a single son; shaded areas show 95% CI. See Supplementary Figure S5 for means and SE. The sample size of fathers across the six PAC treatments (from 4 to 74 days) is 25, 25, 26, 26, 37, and 5, respectively.

In our first approach, we tested for the effects of PAC on lifespans of sons without accounting for the effects of paternal lifespan. We found a significant positive effect of PAC on the lifespans of sons (*t* = 2.532; *p* = 0.013; Supplementary Table S5; Figure 5; Supplementary Figure S5), even when the 74-day-old PAC treatment was excluded (*t* = 2.983; *p* = 0.004). Lifespans of sons increased by ~0.1 days with each day of increase in PAC. The number of offspring produced by fathers had no effect on the lifespans of sons (*t* = 0.054, *p* = 0.957). In our second approach, we tested for the effects of PAC on residuals from a model that first tested for the effects of paternal lifespans on lifespans of sons. Here, we found no significant effects of PAC on these residuals (*t* = 1.532; *p* = 0.129), including in our sensitivity analysis where data of sons from the 74-day-old PAC treatment were excluded (*t* = 1.923; *p* = 0.057). In our third approach (Supplementary Figure S6; Supplementary Table S6), our path analysis suggested significant direct effects of PAC on sons’ lifespan (*z* = 2.443, *p* = 0.015) and on paternal lifespan (*z* = 4.662, *p* < 0.001). This path analysis also revealed marginally nonsignificant effects of paternal lifespan on sons’ lifespan (*z* = 1.920, *p* = 0.055), as well as marginally nonsignificant indirect effects of PAC on sons’ lifespans via the effect of paternal lifespan (*z* = 1.776, *p* = 0.076). Results from our sensitivity analysis, where sons belonging to the 74-day-old PAC treatment were excluded, showed qualitatively similar results. Specifically, increasing PAC significantly increased paternal (*z* = 4.245, *p* < 0.001) and sons’ lifespans (*z* = 2.723, *p* = 0.006); paternal lifespan positively correlated with lifespan of sons (*z* = 1.994, *p* = 0.046); and there was a trend for PAC to influence sons’ lifespans via the indirect effect of PAC on paternal lifespans (*z* = 1.808, *p* = 0.071). Collectively, our results for aim 3 indicate that the positive effects of advancing PAC on the lifespans of sons is partly due to older fathers living longer. This result is in line with the viability selection hypothesis rather than Lansing effects and is not due to trade-offs between paternal reproductive output and sons’ lifespans.

## Discussion

PAC can affect paternal survival and reproductive success, and the fitness of offspring in complex ways, with several hypothesized outcomes. Studying the effects of paternal age at a population level, therefore, requires simultaneously testing for multiple, nonmutually exclusive processes (e.g., reproductive senescence, selective disappearance, terminal investment, Lansing effect, viability selection, trade-offs). Currently, there is a scarcity of studies adopting such an approach. We used experiments in fruit flies to investigate how PAC affects the survival (aim 1) and reproductive output (aim 2) of fathers and the lifespans of their sons (aim 3) and show that the effects of PAC are more complex than predicted by any single hypothesis (see Supplementary Appendices 1 and 2 for predictions for each hypothesis).

### Aim 1: Effects of PAC on paternal survival

We found that while increased PAC was associated with higher paternal lifespans, mating itself was related to increased mortality in older fathers. Mating is known to be energetically and physiologically costly to individuals and can often reduce overall lifespan (Heinze, 2016; Kemp & Rutowski, 2004; Koppik et al., 2018; Partridge & Andrews, 1985; Paukku & Kotiaho, 2005; Perry & Tse, 2013; Sanghvi et al., 2021; Scharf et al., 2013). We found no differences in the overall survival of unmated males and that of the fathers who mated when young. However, we found that fathers who mated at older ages had higher mortality than fathers who mated when younger (see Clinton & Le Boeuf, 1993, who show the opposite in seals), even after removing the effects of actuarial senescence, which has not been previously reported. This result is likely due to old males being frailer and physiologically more vulnerable than young males (e.g., Bisset & Howlett, 2019; Minois & LeBourg, 1999), such that the cost of mating affects the survival of old males more than young males.

### Aim 2: Effects of PAC on paternal reproductive output

We found significant interactive effects of paternal lifespan and PAC on paternal *p*_*s*_ (the probability of siring an offspring). Specifically, paternal lifespan covaried positively with paternal *p*_*s*_, but only for fathers who conceived at older ages. This result could be because most (> 92%) males in the three youngest PAC treatments sired offspring, and there was little variance in *p*_*s*_ for younger PAC treatments. However, infertility became more apparent in older PAC treatments, leading to more variance in *p*_*s*_ for older PAC treatments. Therefore, positive covariances (that are less apparent when there is lower variance in dependent variables) between paternal lifespans and *p*_*s*_ in old but not young PAC treatments might be due to statistical effects rather than true biological effects. While the covariance between lifespans and *p*_*s*_ could have also been negative, a positive covariance would be expected if reproductive output is condition-dependent (Bonduriansky & Brassil, 2005; Chen et al., 2016; Sultanova et al., 2020). Thus, for an older male, ceasing of offspring production could be a reliable indication of the individual’s proximity to death. However, for young males this would not be so, because of low variance in *p*_*s*_. Similarly, this result might indicate that the lifespans of older, but not younger males might be a reliable cue of their reproductive quality (Brooks & Kemp, 2001; Johnson & Gemmell, 2012). Future studies could investigate whether these patterns hold true, in systems where variance in reproductive success is the same for old and young males.

We found no evidence for the terminal investment hypothesis. Specifically, older males who died soon after mating produced fewer offspring than older males who died later or than young males. This result is inconsistent with some studies in birds and insects that have found older males (Duffield et al., 2018; Farchmin et al., 2020; Velando et al., 2006) and females (Creighton et al., 2009; Froy et al., 2013; Part et al., 1992) to invest terminally in reproduction. However, males in many of these studies were also immune-challenged (reviewed in Duffield et al., 2017), which can cause individuals to perceive themselves as being diseased, which was not the case in our study. Moreover, all fathers in our study only mated once. If males perceive their remaining reproductive opportunities based on the number of mates previously encountered, then the lack of variance in the number of matings for each father imposed by our study could have eliminated variation in the perception of residual reproductive opportunities for fathers, thus preventing terminal investment. When averaged across the effects of male age, we found no evidence for paternal lifespan covarying positively with the number of offspring sired by a male (*N*_*s*_). While we found evidence for longer-lived fathers having a higher probability of producing offspring, this result was significant only in older PAC treatments. Evidence for selective disappearance would require detecting positive covariances between paternal lifespan and *N*_*s*_ or *p*_*s*_ across all PAC treatments. Our results, therefore, do not support selective disappearance occurring across all PAC treatments in our study.

Our results showed some evidence for reproductive senescence (also seen in other fruit fly studies: Grotewiel et al., 2005; Ruhmann et al., 2018; Sepil et al., 2020). Reproductive senescence in our study was driven by old males (46 days and older) being less likely to sire an offspring and the oldest PAC treatments (i.e., 74 days old) siring the fewest numbers of offspring. Overall, our results suggest that male reproductive output increases from mid- to adult life, and reproductive senescence onsets in only much later in adult life (Baudisch & Stott, 2019; Jones et al., 2014; Sanghvi et al., 2024). Future studies should investigate the extent to which the patterns of reproductive senescence observed in our study are due to declines in sperm number (Gasparini et al., 2010, 2019; Sepil et al., 2020), sperm performance (Dean et al., 2010), seminal fluid quantity (Reinhardt et al., 2011), or changes in seminal fluid composition (Fricke et al., 2023). In our study, PAC correlated with the period for which fathers remained sexually and socially isolated, therefore, we cannot disentangle the role of period of isolation on paternal reproductive output.

### Aim 3: Effects of PAC on lifespans of sons

Older fathers produced longer-lived sons, a result that is not in line with the Lansing effect (Crow, 2003; Lansing, 1947; Monaghan et al., 2020; Sharma et al., 2015; Wylde et al., 2019). Our results contrast with other studies in fruit flies that show a Lansing effect (e.g., Mossman et al., 2019; Price & Hansen, 1998), whereby offspring born to older fathers have lower survival. There could be several reasons for this discrepancy.

First, fathers in our experiment were kept individually isolated, without exposure to rival males, whereas Mossman et al. (2019) and Price and Hansen (1998) kept males in single-sexed groups. Exposure to rivals has been shown to cause *Drosophila* males to invest more in sperm production (Bjork et al., 2007; Hopkins et al., 2019) at the cost of reduced investment in maintaining sperm quality (Koppik et al., 2023; Silva et al., 2019) compared to males kept individually. Thus, it is possible that fathers in our experiment would have been able to prevent deleterious paternal age effects mediated via deterioration in sperm quality due to being kept individually. Second, the lack of exposure to rival males in our study could have reduced the rate of sperm production, thus, germline cell division and mutation rates in fathers (Crow, 2003; de Manuel et al., 2022; Girard et al., 2016; Monaghan & Metcalfe, 2019), compared to studies showing Lansing effects that kept males in groups. This possible reduction in germline mutation rate could further buffer the effects of paternal age. Third, sons in our experiment were not mated. Life-history theory predicts trade-offs between allocating energy to somatic maintenance versus reproduction (Lemaître et al., 2015; Stearns, 1989). Being kept unmated could have allowed sons to invest in somatic maintenance, possibly masking deleterious paternal age effects, and future studies could test this by repeating our experiments but introducing another treatment where sons mate. Fourth, we sampled fathers at extremely old lifespans (i.e., up to 74 days, representing > 75% of maximum lifespan). However, studies that have shown deleterious paternal age effects in *D. melanogaster* sampled “old” fathers between 14 and 45 days of age (e.g., Mossman et al., 2019; Nystrand & Dowling, 2014; Price & Hansen, 1998; Sepil et al., 2020). It is possible that at ages as extreme as in our study, the deleterious effects of PAC are outweighed by the positive effects of selection on paternal viability. Fifth, we sampled fathers cross-sectionally to create heterogeneity in paternal lifespans between PAC treatments because we were explicitly interested in testing the viability selection hypothesis (Beck & Powell, 2000; Kokko, 1998). However, this sampling could have masked within-individual deterioration of sperm, thus the Lansing effect, which might be better revealed when fathers are sampled longitudinally.

Our results provided some support for the viability selection hypothesis (Brooks & Kemp, 2001; Hansen & Price, 1995; Johnson & Gemmell, 2012; Kokko, 1998). Specifically, fathers that mated when older had longer lifespans, and produced sons with longer lifespans. When effects of paternal lifespans on sons’ lifespans were removed (i.e., second statistical approach), PAC no longer had a significant effect on the lifespans of sons. These results suggest that significant positive effects of PAC on lifespans of sons were driven by paternal lifespans. There could, however, be other mechanisms that could explain the direct effect of PAC on the lifespans of sons. For instance, poor-quality offspring of old fathers could have experienced death at the larval or pupal stage, thus eclosed sons representing a biased sample of high-quality offspring (e.g., Sanghvi et al., 2022). Additionally, a sample size of three sons per father might not have been representative of the average lifespans of sons, which could have increased noise in our results. Unlike Johnson et al. (2018), we found no evidence that the increased lifespans of sons from old males were due to trade-offs between paternal investment in reproductive output versus investment in offspring quality (i.e., increased lifespan of sons). Our results overall suggest that increasing PAC selects for fathers that have alleles that confer higher viability, which are then inherited by the sons of older fathers. Some previous studies have found positive effects of PAC on offspring lifespans (Angell et al., 2022; Johnson et al., 2018; Krishna et al., 2012; Lee et al., 2019; Priest et al., 2002). However, to our knowledge, ours is the first to formally test the viability selection hypothesis by investigating separately the direct as well as indirect effects (via paternal lifespans) of PAC, on offspring lifespans. It is possible that sons from older fathers have longer lifespans but lower reproductive output (e.g., Long & Pischedda, 2005) or that paternal age effects are sex-specific (Angell et al., 2022; Bouwhuis et al., 2015; Priest et al., 2002; Schroeder et al., 2015; Sparks et al., 2022), something future studies ought to investigate. Collectively, our results indicate that being born to old fathers need not be deleterious to the offspring lifespan.

## Conclusions

Our study simultaneously tests various mechanisms that may link PAC to paternal survival (age-dependent frailty), reproductive output (reproductive senescence, selective disappearance, terminal investment), and offspring lifespans (Lansing effect, viability selection, trade-offs between paternal investment in offspring quality versus quantity). We show that mating at older ages can lead to an increased risk of mortality for fathers, thus buffering the magnitude of positive covariances between PAC and paternal lifespans. We further reveal that positive pleiotropy between survival and reproductive output depends on the age of the father. We also find that senescence in reproductive output occurs late in life, and its onset differs for the likelihood of producing offspring versus the number of offspring produced. Lastly, we show that advancing PAC has a positive effect on the lifespans of sons and that this effect is likely due to older fathers having longer lifespans. Our study highlights that demographic processes such as differential mortality risk and viability selection can mask possible within-individual deterioration in reproduction. We thus recommend that future studies employ a broader theoretical framework that weighs the costs of reproducing at an old age against its direct and indirect benefits to better understand organismal health and life history. Fitness benefits provided by old fathers producing longer-lived sons might offset the fitness costs of reduced fertility in old fathers. If so, this offset could lead to the evolution of female preference for old males (Beck & Powell, 2000; Beck & Promislow, 2007; Beck et al., 2002; Johnson & Gemmell, 2012; Kokko & Lindström, 1996) and future studies can investigate this exciting but relatively unexplored avenue of research.

## Supplementary Material

Supplementary Material

## Data Availability

Data and all associated code are available on OSF with DOI: 10.17605/OSF.IO/38CXN at https://osf.io/38cxn/ and on DRYAD at https://doi.org/10.5061/dryad.80gb5mkzm.
