# Supplementary material for: What does not kill you makes you stronger? Effects of paternal age at conception on fathers and sons
Source: Evolution. Author manuscript; Available in PMC 2025 Feb 24. (PMC7617388; doi:10.1093/evolut/qpae097)
Supplement: Supplementary Material [file EMS199600-supplement-Supplementary_Material.pdf]

Supplementary information

Appendix 1: Effects of paternal age on paternal survival (hypotheses and predictions)

It is commonly assumed that as age at conception increases, lifespan should increase, i.e., fathers who mate at an older age should have higher lifespans (H1A). A corollary prediction is that at older age groups, there should be lower variances in lifespan. These predictions are based on the fact that individuals who mate at an older age have a lower limit for what lifespans they can have, because they have by definition survived to the age at which they conceive. For instance, if males mate at age 60 days, all males have survived until at least 60 days of age. If all individuals have a similar upper limit to lifespans, irrespective of the age at which they mate, then older ages of conception would have lower variance but higher means in lifespans. This leads to the effects of age at conception on lifespan to look as follows (Figure A):

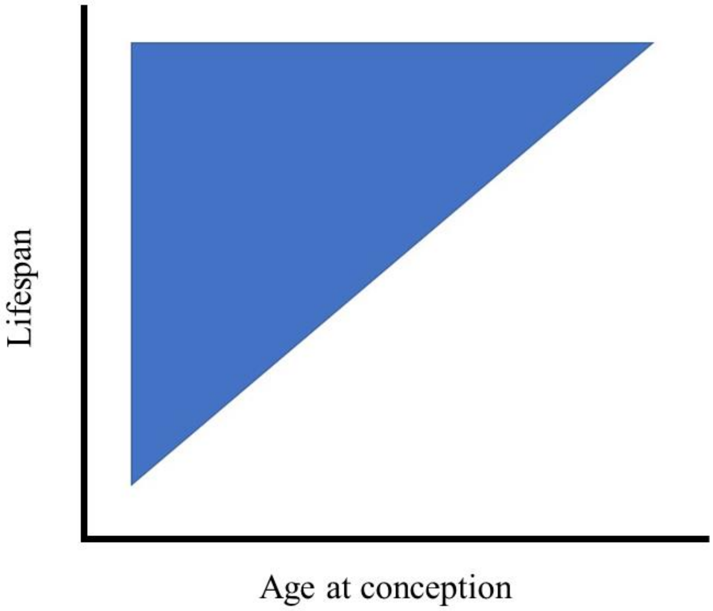

Figure A: Advancing age at conception leads to higher means but lower variance in lifespan of older individuals

However, this prediction has two assumptions which are not usually explicitly tested. The first assumption is that individuals in a population start dying from birth onwards. Thus, all age classes at which individuals conceive offspring, would have experienced some mortality, even if the rate of mortality would only increase after maturity. However, it is possible that death in a population does not occur until a certain age (e.g. if survival curves are sigmoid shaped, and risk of mortality only increases after a certain age). Here, the lower limit for lifespans for various ages at conception might be the same. For instance, if in a population, males mate at ages 10, 20, 30, 40 …. to 90 days of age, but no males die until 40 days of age, then for males who mate at ages 10, 20, 30, and 40 days, the bottom limit of lifespan will be 40 days of age. This can lead to effects of age at conception on lifespan to look as follows (Figure B):

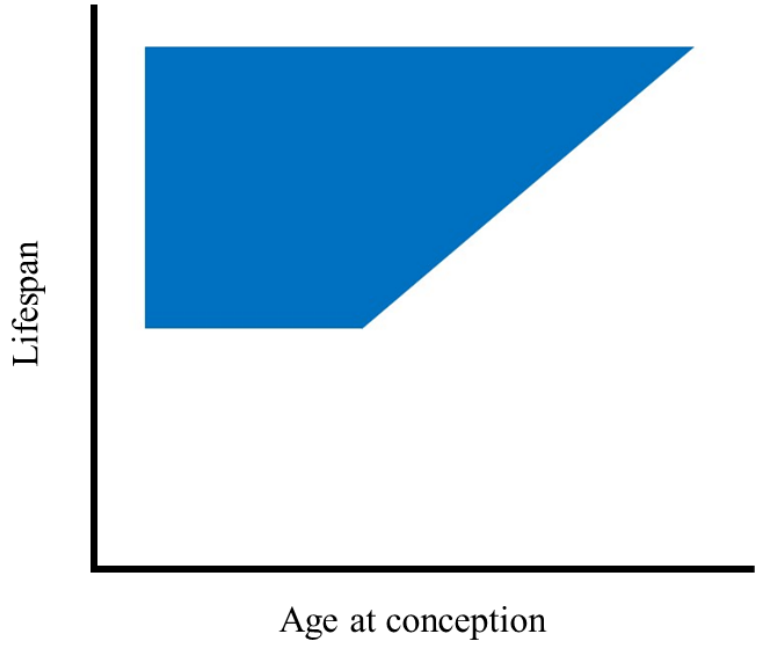

Figure B: Advancing age at conception leads to higher means but lower variance in lifespan of older individuals, however, mortality does not occur until a certain age, thus age at conception groups until that age have the same average lifespans.

The second assumption is that the upper limit for lifespans across all ages of conception is the same. That is, irrespective of at what age individuals reproduce, they have the same probability of surviving until the upper limit of lifespan for that population. This however might not always be true. For instance, if older males are frailer than younger males, males mated when old might have a lower ceiling for longevity. An example of this is if mating related stress increases mortality in males who are more vulnerable (e.g. old mated males), thus reduces lifespans of males who mate when old but not when they mate young. This can lead to effects of age at conception on lifespan to look like Figure C.

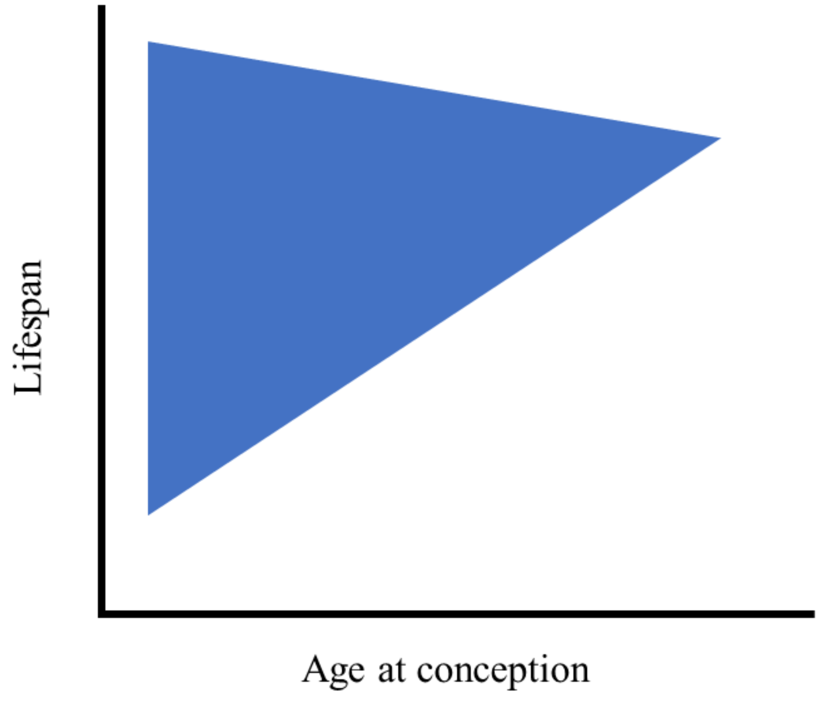

Figure C: Advancing age at conception leads to higher means but lower variance in lifespan of older individuals. However, males who mate when older experience higher mating-related stress due to being frailer.

Combining the effects of assumption 1 and 2, we get a complex relationship between age at conception and lifespan (Figure D), which is quite different from the commonly held prediction in Figure A. What this complex relationship would look like in our data is presented in Figure E.

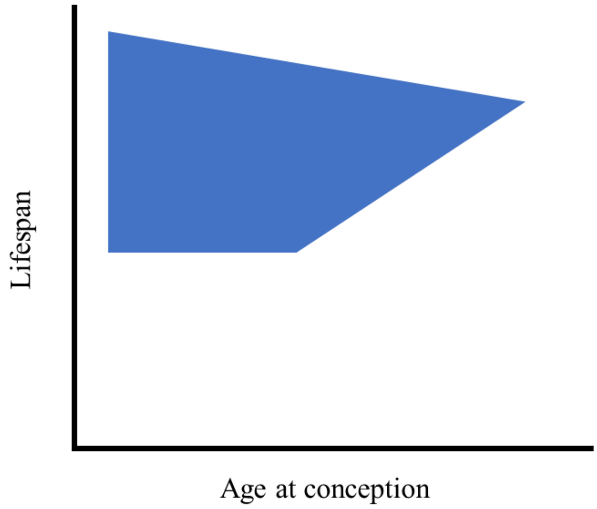

Figure D: Advancing age at conception leads to higher means but lower variance in lifespan of older individuals. However, males who mate when older, experience higher mating-related stress due to being frailer, and mortality does not occur until a certain age, thus age at conception groups until that age have the same average lifespans. This leads to a complex relationship between age at conception and lifespan.

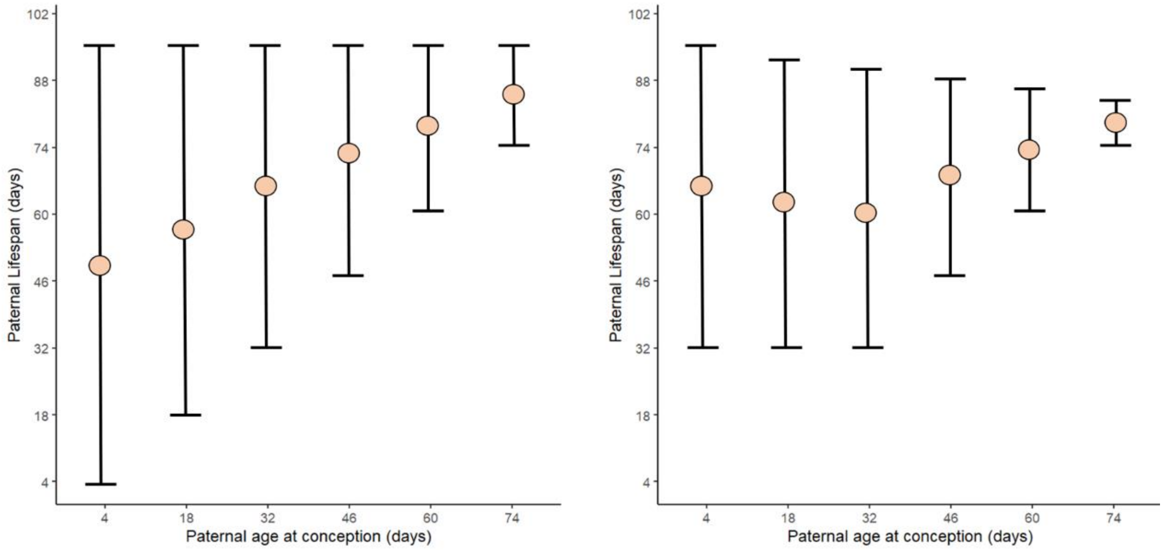

Figure E: (Left) Based on our experimental design, if there is no age-dependent frailty or late onset of death, then advancing paternal age at conception leads to a linear increase in lifespans of fathers. (Right) However, if males who mate when older experience higher mating-induced mortality due to being frailer (creating a lower ceiling for lifespan with increasing PAC), and if death does not occur until a certain age (say 32 days), then a non-linear relationship between PAC and paternal lifespan ensues. Range of (max-min), and mean lifespans for, each PAC group shown, assuming that the mean is the middle value of the range.

Appendix 2: Graphical predictions for different hypotheses in each of the three aims, for how paternal lifespan, PAC, paternal reproductive output, and offspring lifespans, might be linked.
Note that presented graphs are hypothetical

Aim 1: Effects of PAC on paternal survival
<u>Age-dependent frailty hypothesis</u>
Prediction: If mating does not affect survival of males, then unmated males, and males who mate when young or when old, should all have same lifespans.

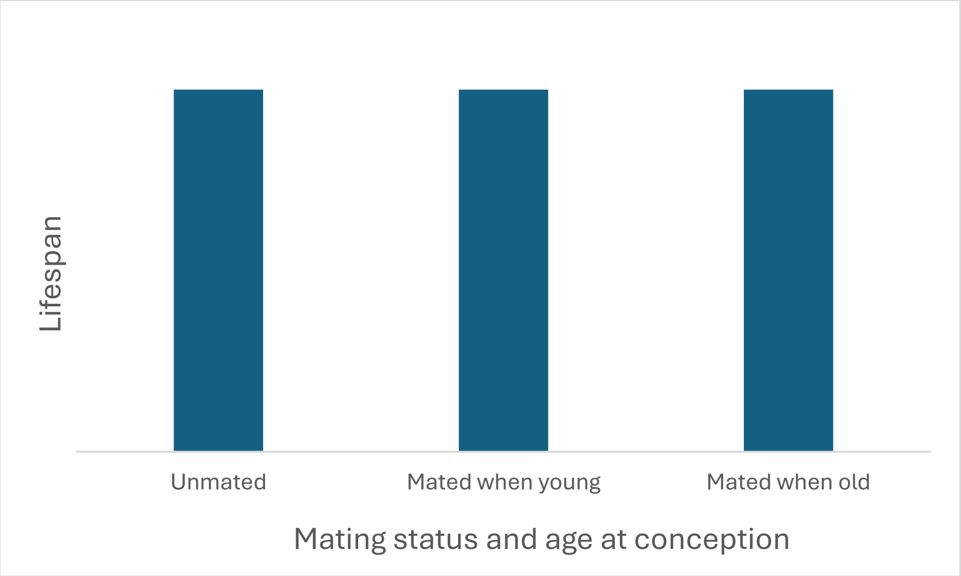

Figure F: No effects of age and/or mating status to affect lifespan.

If mating-stress reduces the survival of males irrespective of the age at which males mate, then unmated males should have higher lifespans than mated males.

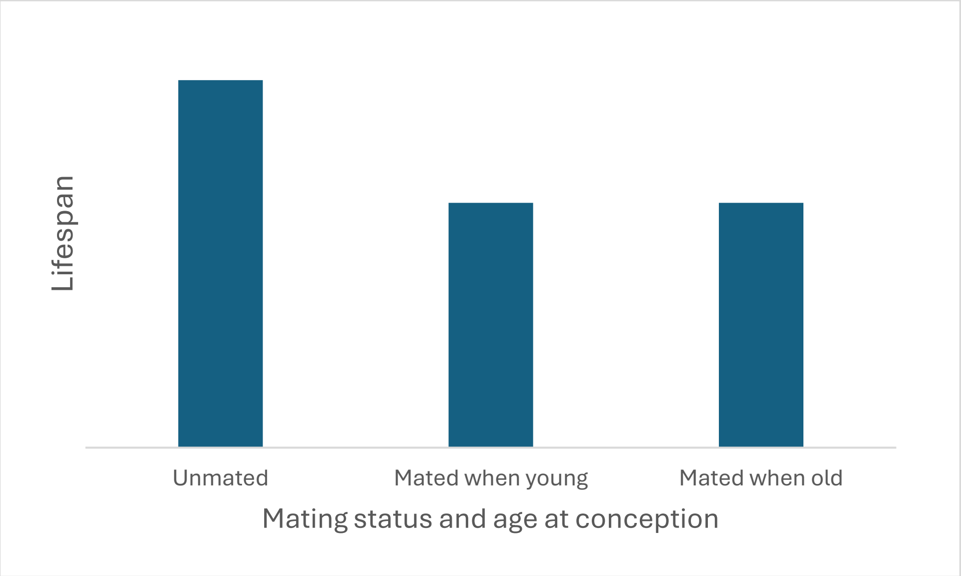

Figure G: Mating status, but not age, affects lifespan, with unmated males living longer.
However, if old are vulnerable to mating stress due to frailty, then mating-related stress should reduce the survival of males who mate when old, but not when young.

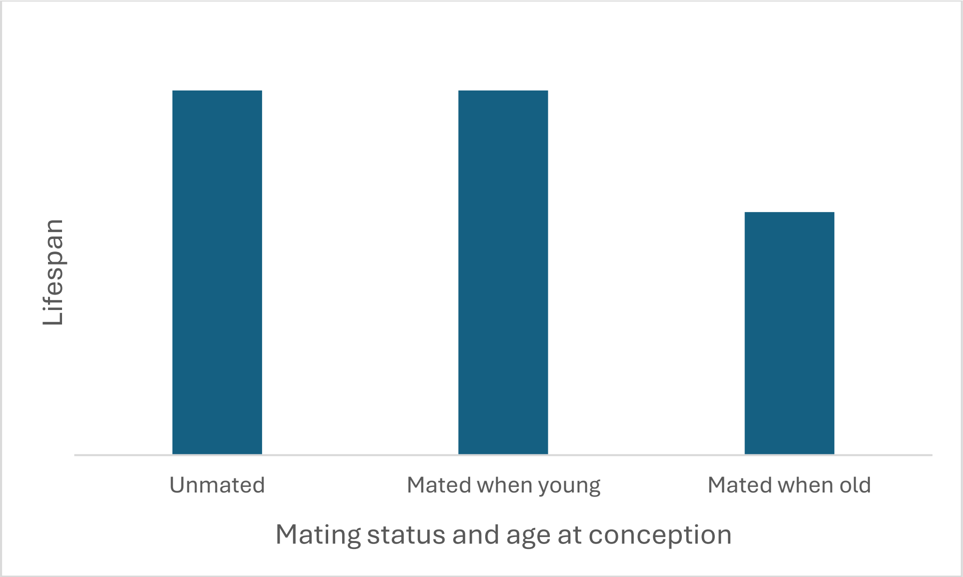

Figure H: Mating status interacts with age to affect lifespan, with mated, older males living the shortest.

Aim 2: Effects of PAC on paternal lifespan

Reproductive senescence hypothesis

Prediction: If reproductive senescence occurs, old males should have lower reproductive output than young males.

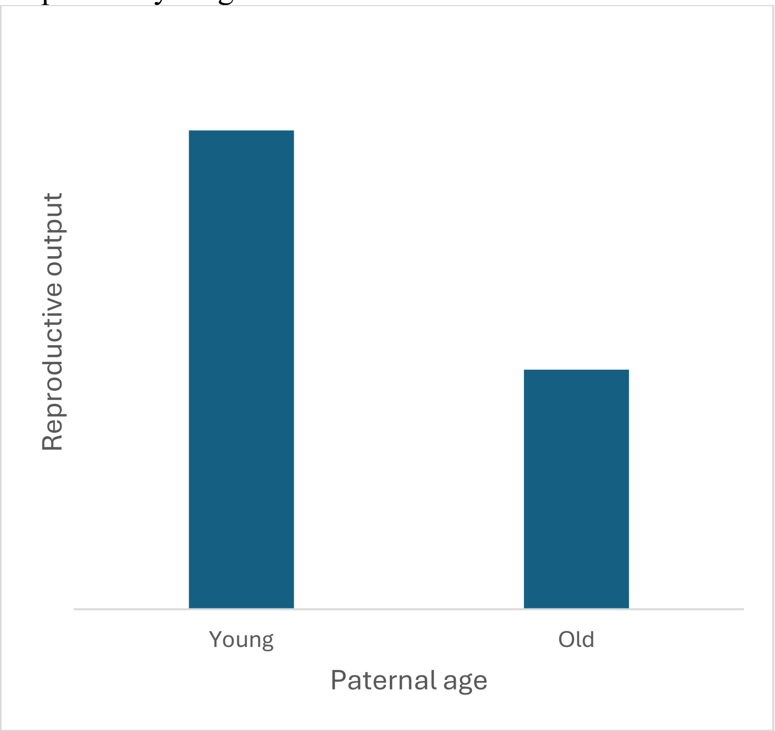

Figure I: Old males have lower reproductive output than young males due to reproductive senescence

Selective disappearance hypothesis

Prediction: Males who produce fewer offspring will selectively disappear (i.e. die) with age, leading to population level increases in reproductive output with advancing male age. Younger age groups will contain males of poor and high reproductive output while older age groups will contain males of only higher reproductive output, thus have lower variance in reproductive output.

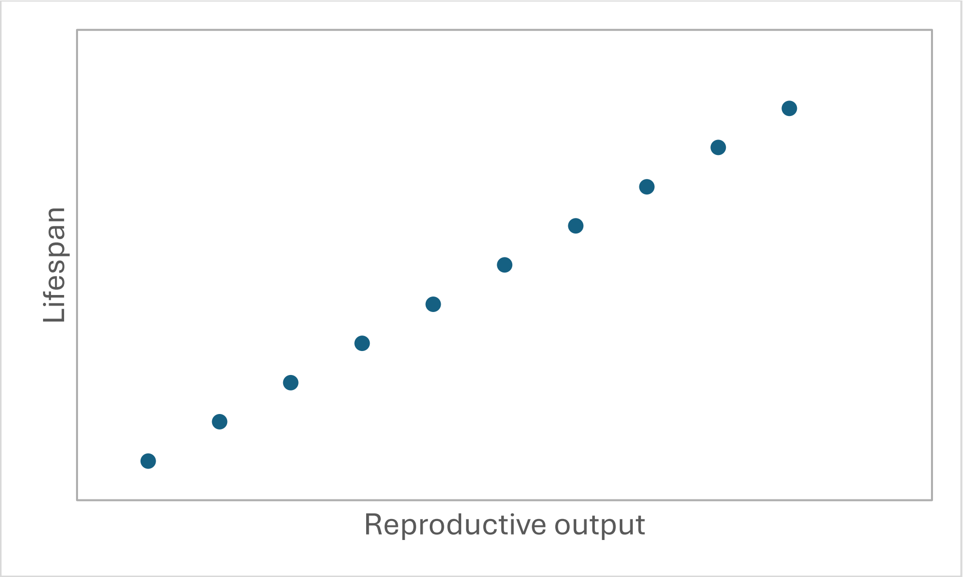

Figure J: Reproductive output and lifespan co-vary positively due to positive pleiotropy between life-history traits

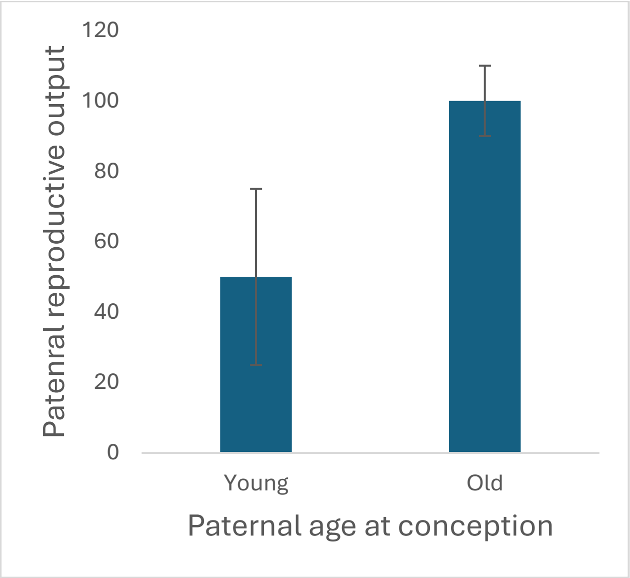

Figure K: Positive covariances between reproductive output and survival leads to selective disappearance of males and old males having higher reproductive output but lower variance in reproductive output, than young males

Dynamic terminal investment hypothesis

Prediction: Older males who are close to dying should invest more in reproduction than young males, or than old males who are not close to dying.

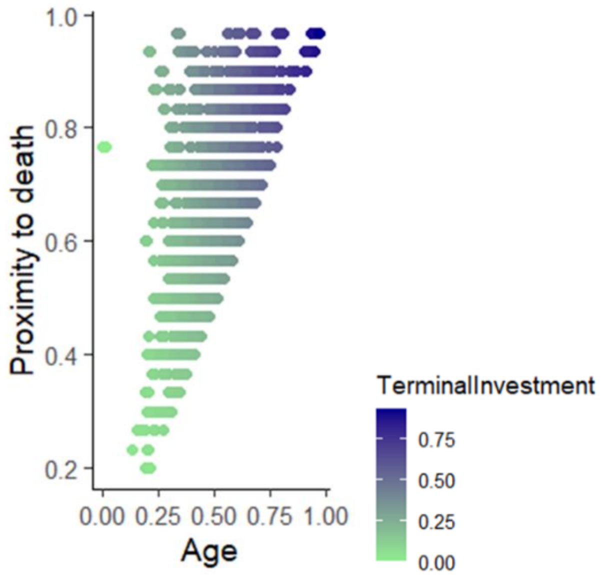

Figure L: Age at conception and proximity to death would interact to influence likelihood of terminal investment. Here, old males who are about to die soon would terminally invest in reproductive output, and have higher reproductive output, than old males who are not about to die soon, or than young males.

Aim 3: Effects of PAC on sons' lifespans

<u>Lansing effect hypothesis</u>

Prediction: Old fathers produce sons with shorter lifespans

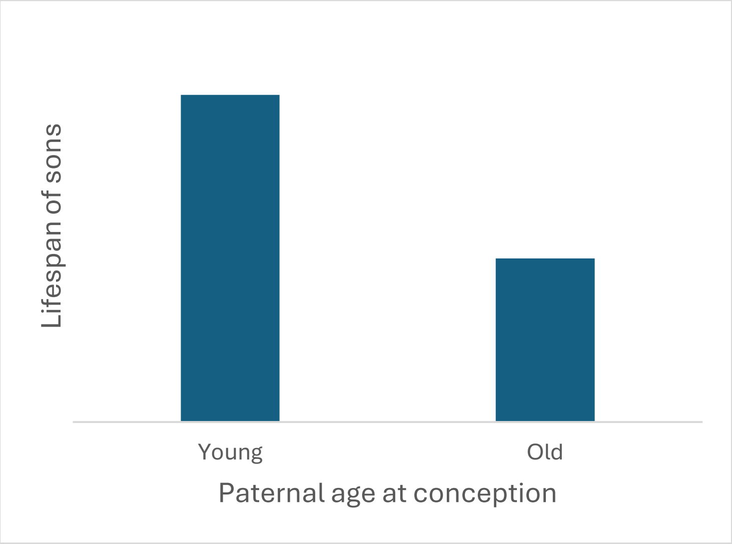

Figure M: Old father produce sons with lower lifespans due to Lansing effect

<u>Viability selection hypothesis</u>

Prediction: Fathers who mate at older ages are on average longer lived (however have lower variances in lifespan), and produce sons with longer lifespans (but lower variances in lifespan).

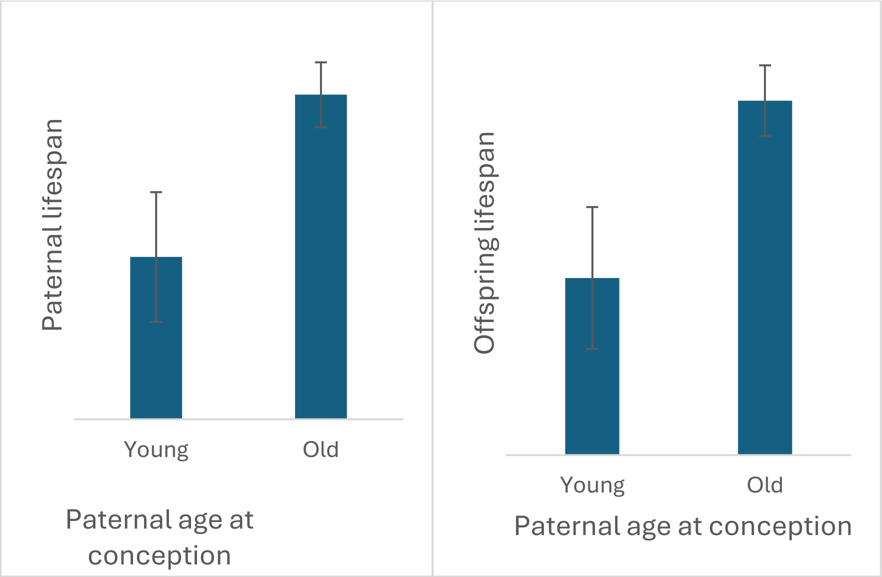

Figure N: Old fathers have longer lifespans but lower variance in lifespans (also corresponding to H1A), and produce sons with longer lifespans but lower variance in lifespans, than young fathers.

Appendix 3: Tables and model outputs

Table S1: Sample sizes for number of males who were mated at a particular age, and from these, the number of males who produced offspring. Note that at age 60 days, a higher number of males were selected to mate because of the high rate of infertility of old males. Additionally, at 74 days of age, very few males were surviving in the unmated stock population, thus the low sample size.

PAC (days)	N mated	N produced offspring
4	25	25
18	25	23
32	26	25
46	26	15
60	37	14
74	5	2

Table S2: Model output for effects of paternal age at conception (PAC) on probability of surviving three days after mating, with paternal offspring production (F1_count) included as a covariate. Terms highlighted in grey used for interpretation of interaction and main effects.

	Estimate	SE	z	P
(Intercept)	7.726	2.176	3.551	<0.001
PAC	-0.131	0.037	-3.544	<0.001
F1_count	0.018	0.010	1.764	0.078

Table S3: Hurdle model to test interactive effects of paternal age at conception and paternal lifespan, on reproductive output of fathers. Zero inflation model tests effects on the probability of not producing an offspring. Conditional model tests effects on the number of offspring produced, when only data on fathers that produced an offspring were analysed. PAC = paternal age at conception, Paternal_LS = paternal lifespan. Terms highlighted in grey used for interpretation of interaction and main effects.

Two-way interaction model:

Dispersion parameter	6.19

Conditional model (>0)	Estimate	SE	z	P
(Intercept)	3.928	0.284	13.819	<0.001
PAC	0.011	0.011	1.021	0.307
Paternal_LS	0.001	0.004	0.126	0.899
I(PAC^2)	0.000	0.000	-3.870	<0.001
Latency	0.000	0.001	-0.092	0.927
Copulation duration	0.006	0.007	0.951	0.342
PAC:Paternal_LS	0.000	0.000	0.562	0.574

Zero-inflation model (1 vs 0)	Estimate	SE	z	P
(Intercept)	-9.046	5.040	-1.795	0.073
PAC	0.349	0.129	2.703	0.007
Paternal_LS	0.094	0.071	1.312	0.190
I(PAC^2)	0.000	0.001	0.270	0.787
Latency	-0.029	0.012	-2.438	0.015
Copulation duration	-0.089	0.046	-1.937	0.053
PAC:Paternal_LS	-0.004	0.002	-2.336	**0.020**

Main-effects model:

Conditional model (>0)	Estimate	SE	z	P
(Intercept)	3.829	0.226	16.921	<0.001
Latency	0.000	0.001	-0.189	0.850
Copulation duration	0.006	0.007	0.882	0.378
Paternal_LS	0.002	0.002	0.963	0.336
PAC	0.016	0.006	2.598	**0.009**
I(PAC^2)	0.000	0.000	-3.902	**<0.001**

Zero-inflation model (1 vs 0)	Estimate	SE	z	P
(Intercept)	-0.621	2.335	-0.266	0.790
Latency	-0.023	0.010	-2.229	0.026
Copulation duration	-0.101	0.043	-2.340	0.019
Paternal_LS	-0.065	0.026	-2.526	0.012
PAC	0.192	0.092	2.081	0.037
I(PAC^2)	-0.001	0.001	-0.993	0.321

Table S4: Effects of paternal age at conception and days to death (i.e. time elapsed between death and mating) on the number of offspring produced by fathers. PAC = paternal age at conception. Terms highlighted in grey used for interpretation of interaction and main effects.

Two-way interaction model	Estimate	SE	z	P
(Intercept)	4.204	0.096	43.830	<0.001
Days_to_death	-0.004	0.001	-2.700	0.007
PAC	-0.009	0.002	-6.050	<0.001
Copulation duration	0.008	0.003	2.970	0.003
Days_to_death:PAC	0.000	0.000	6.850	**<0.001**

Main-effects model	Estimate	SE	z	P
(Intercept)	4.008	0.253	15.823	<0.001
Days_to_death	0.001	0.003	0.167	0.867
PAC	-0.004	0.003	-1.329	0.184
Copulation duration	0.011	0.008	1.381	0.167

Table S5: Effects of paternal age at conception on lifespans of sons, without accounting for effects of paternal lifespan. Heterogeneous variance function specified as a power function, with parameter showing change in variance in lifespans of sons, with the increasing PAC. PAC = paternal age at conception, F1_count= number of offspring produced by fathers. Terms highlighted in grey used for interpretation of main effects.

Random effects	SD
Paternal ID	4.659
Residual	22.002
Heterogenous variance parameter (power)	-0.24169

Fixed effects	Estimate	SE	DF	t	P
(Intercept)	55.904	3.409	188.000	16.399	<0.001
F1_count	0.002	0.043	98.000	0.054	0.957
PAC	0.108	0.042	98.000	2.532	**0.013**

Table S6: Model output from path analysis (structural equation model), showing the direct and indirect effects (via paternal lifespan) of paternal age at conception, on lifespans of sons. F1_LS = lifespans of sons, PAC = paternal age at conception, Paternal_LS = paternal lifespan, F1_count = number of offspring produced by fathers. Terms highlighted in grey used for interpretation of effects.

	Estimate	SE	z	P
F1_LS ~ PAC (c)	0.094	0.039	2.443	**0.015**
Paternal_LS ~ PAC (a)	0.171	0.037	4.662	**<0.001**
F1_LS ~ Paternal_LS (b)	0.115	0.060	1.920	*0.055*
Covariances:	Estimate	SE	z	P
Paternal_LS ~~ F1_count	3.907	12.102	0.323	0.747
F1_LS ~~ F1_count	-0.718	12.272	-0.059	0.953
Variances:	Estimate	SE	z	P
F1_LS	149.855	12.466	12.021	<0.001
Paternal_LS	145.645	12.116	12.021	<0.001
F1_count	290.526	24.169	12.021	<0.001
Defined Parameters	Estimate	SE	z	P
a*b (indirect effect)	0.020	0.011	1.776	*0.076*
c + a*b (total effect)	0.114	0.037	3.040	**0.002**

Appendix 4: Supplementary figures

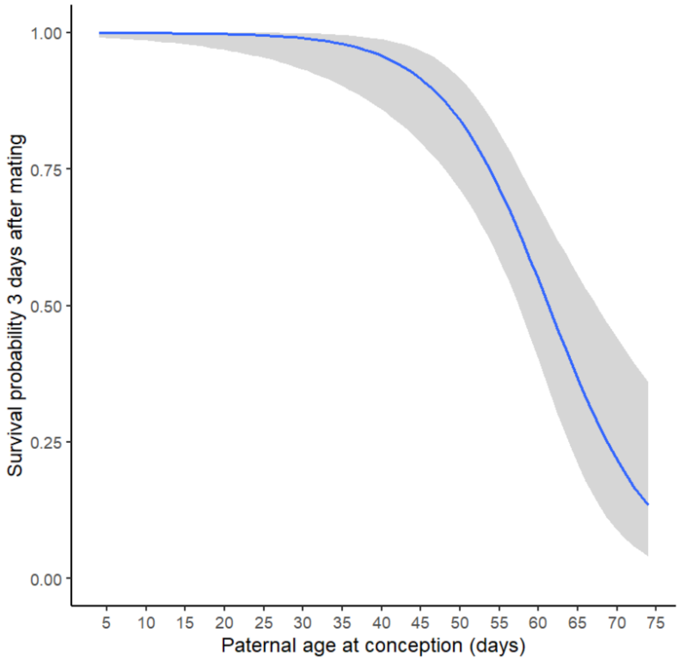

Figure S1: Lower proportion of males are alive 3 days after mating, in older PAC treatments than younger PAC treatments. Shaded areas represent 95% C.I.

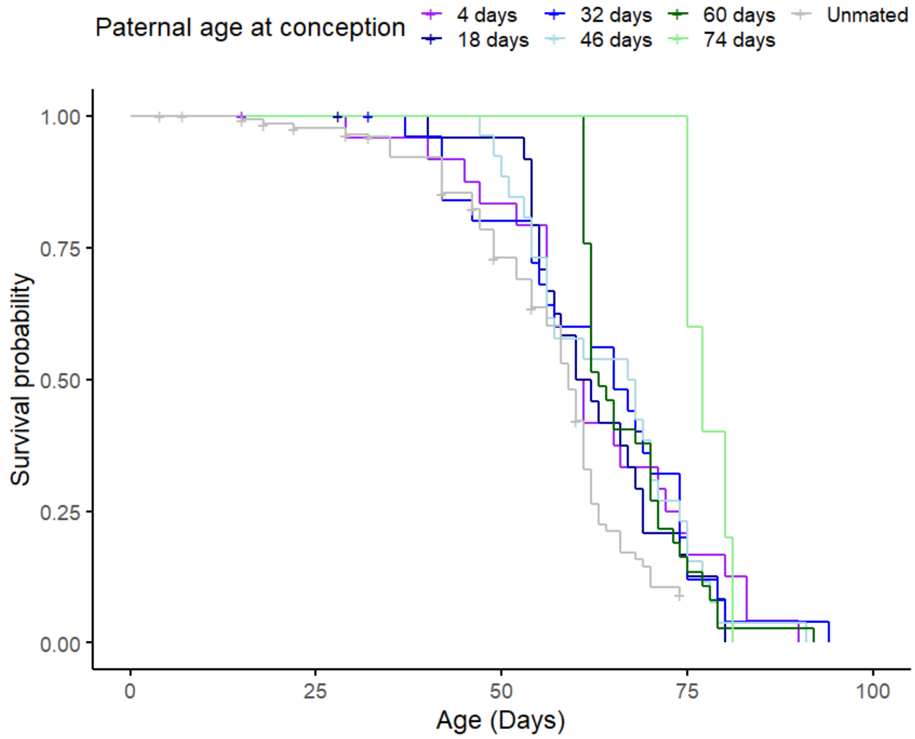

Figure S2: Survival probability of fathers mated at different ages, and males from the unmated experimental population. Fathers who mate at an older age have a lower survival probability at a given age than father who mate at a younger age. "+" signs show censored males.

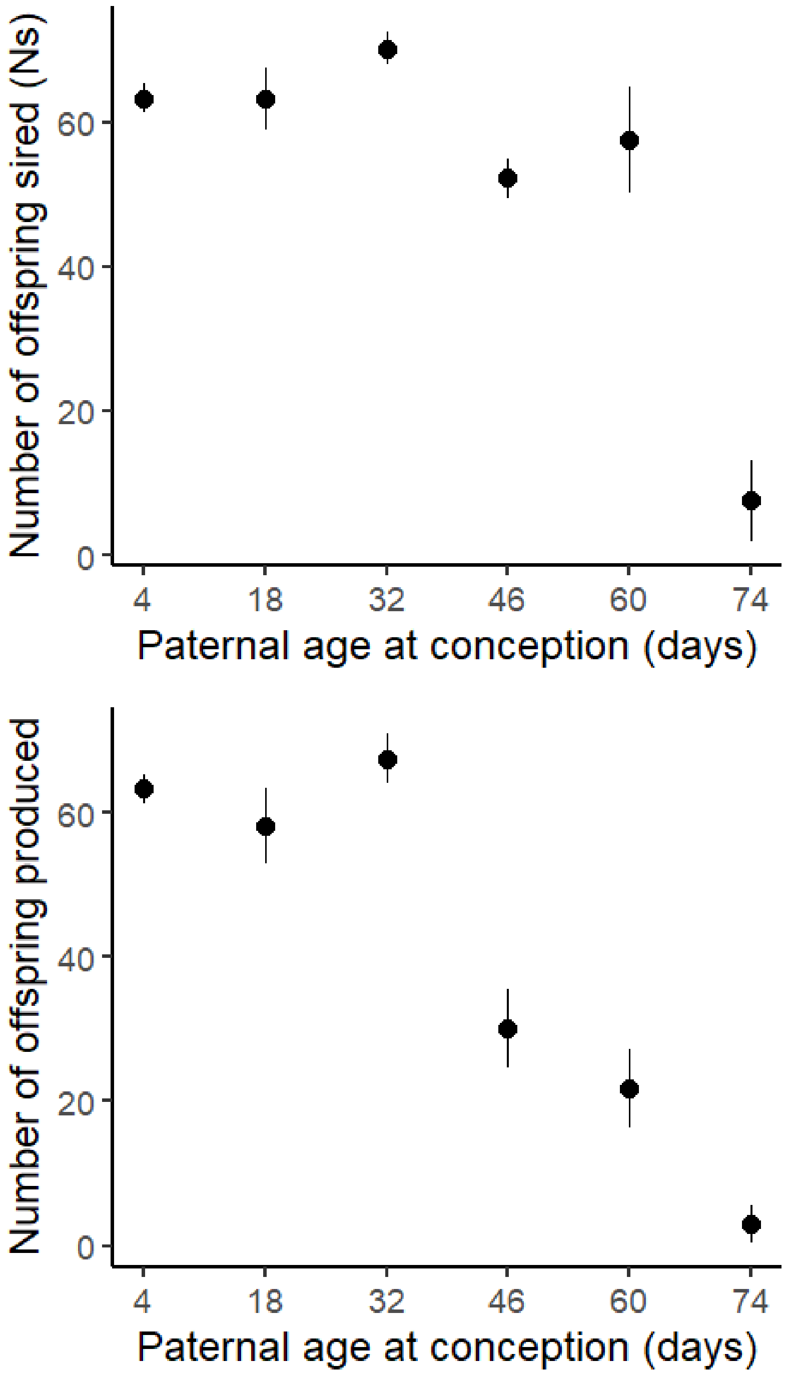

Figure S3: (Top) Paternal age at conception affected the number of offspring sired (excluding fathers who did not produce offspring) in a quadratic way. However, this effect was likely driven by data from PAC of 74 days being lower than other PAC data (also see Figure S4). (Bottom): Effects of paternal at conception on number of offspring produced (including fathers who produced zero offspring). Means and SE shown.

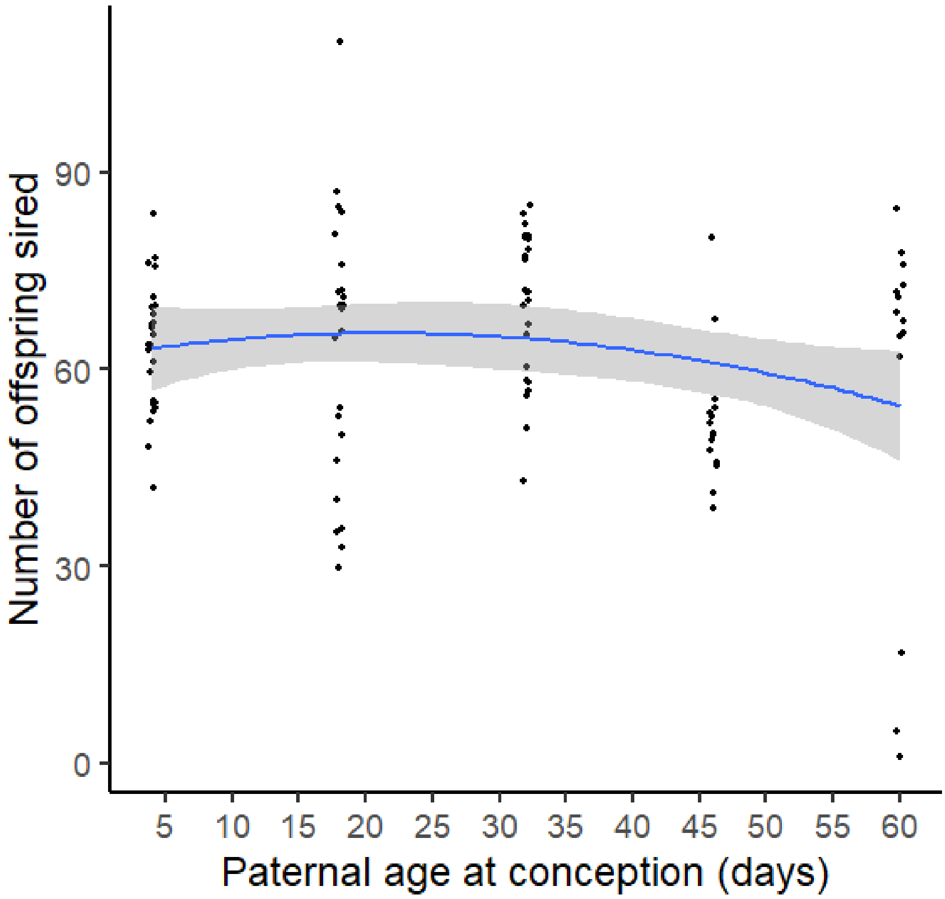

Figure S4: Paternal age at conception affected the number of offspring sired (after excluding fathers who did not produce offspring) in a quadratic way (even when PAC group of 74 days was excluded). However, excluding data from PAC of age 74 days led to a shallow shape of the quadratic curve compared to when this data was included (i.e. Figure 4B). Shaded areas represent 95% C.I.

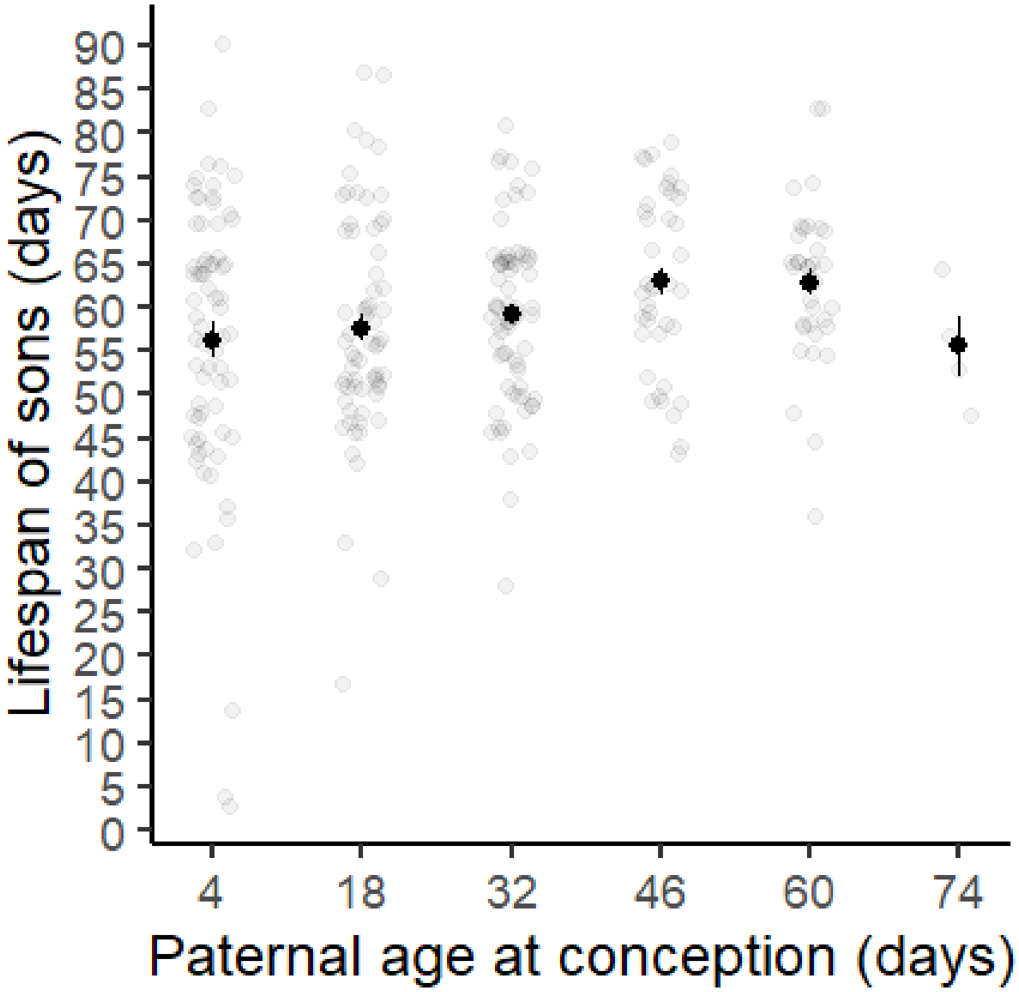

Figure S5: Advancing paternal age at conception increased lifespan of offspring. Means and SE presented

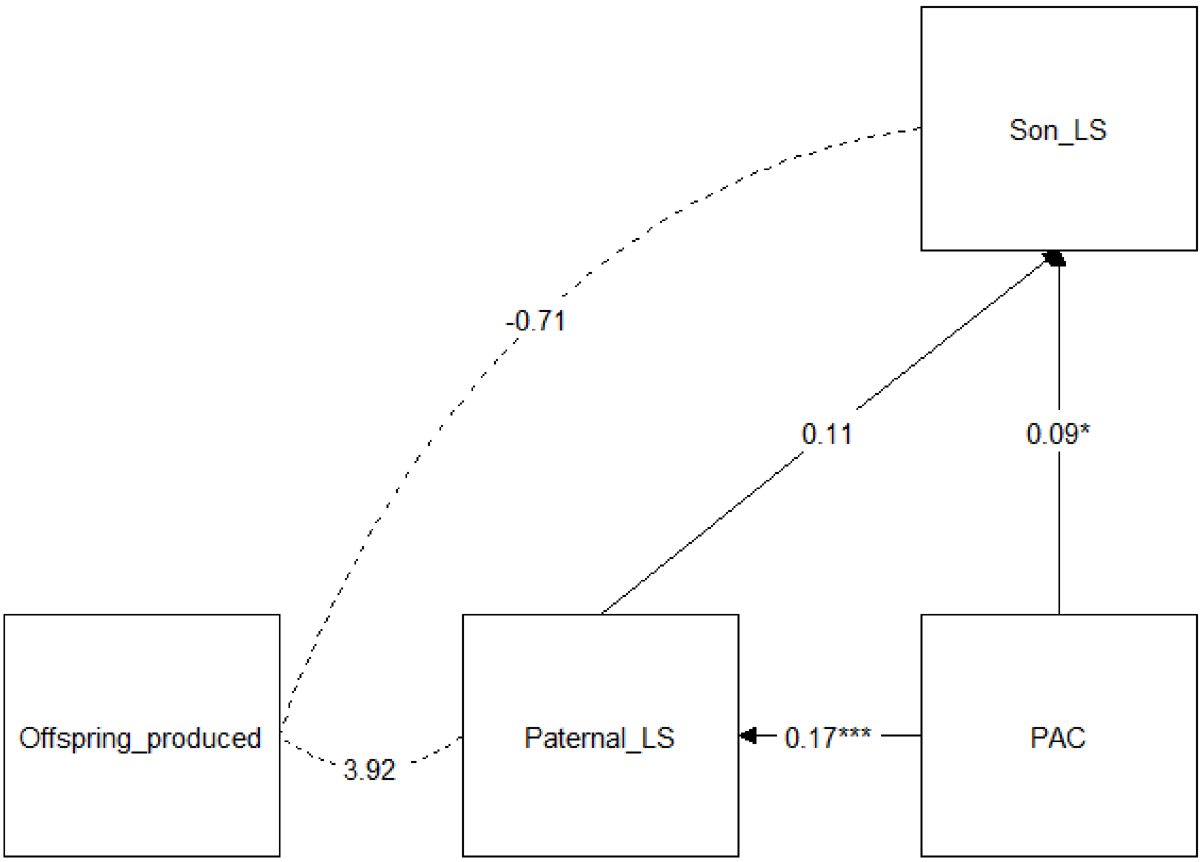

Figure S6: Path analysis showing direct effects of paternal age at conception, and indirect effects via paternal lifespan, on sons' lifespans, along with covariances (dotted) of paternal lifespan, lifespans of sons, and number of offspring produced. Values show estimates of models, and asterisks represent level of significance.
